# PCSK9 Activity Is Potentiated Through HDL Binding

**DOI:** 10.1161/CIRCRESAHA.121.319272

**Published:** 2021-10-04

**Authors:** Sean A. Burnap, Katherine Sattler, Raimund Pechlaner, Elisa Duregotti, Ruifang Lu, Konstantinos Theofilatos, Kaloyan Takov, Gerd Heusch, Sotirios Tsimikas, Carlos Fernández-Hernando, Sarah E. Berry, Wendy L. Hall, Marlene Notdurfter, Gregorio Rungger, Bernhard Paulweber, Johann Willeit, Stefan Kiechl, Bodo Levkau, Manuel Mayr

**Affiliations:** King’s College London British Heart Foundation Centre, School of Cardiovascular Medicine and Sciences, United Kingdom (S.A.B., E.D., R.L., K. Theofilatos, K. Takov, M.M.).; Institute for Pathophysiology, University Hospital Essen, West German Heart and Vascular Center, Germany (K.S., G.H.).; Department of Neurology, Medical University of Innsbruck, Austria (R.P., J.W., S.K.).; Division of Cardiovascular Medicine, University of California San Diego, La Jolla (S.T.).; Vascular Biology and Therapeutics Program, Yale University School of Medicine, CT (C.F.-H.).; Department of Nutritional Sciences, School of Life Course Sciences, Faculty of Life Sciences & Medicine, King’s College London, United Kingdom (S.E.B., W.L.H.).; Department of Internal Medicine, Hospital of Bruneck, Italy (M.N.).; Department of Neurology, Hospital of Bruneck, Italy. (G.R.).; Department of Internal Medicine I, Paracelsus Medical University, Salzburg, Austria (B.P.).; VASCage - Research Centre on Vascular Ageing and Stroke, Innsbruck, Austria (S.K.).; Institute for Molecular Medicine III, Heinrich-Heine-University, Medical Faculty, Düsseldorf, Germany (B.L.).

**Keywords:** apolipoproteins, coronary artery disease, cardiovascular diseases, lipoproteins, mass spectrometry

## Abstract

PCSK9 (proprotein convertase subtilisin/kexin type 9) circulates in a free and lipoprotein-bound form, yet the functional consequence of the association between PCSK9 and HDL (high-density lipoprotein) remains unexplored.

**Objective:**

This study sought to interrogate the novel relationship between PCSK9 and HDL in humans.

**Methods and Results:**

Comparing lipoprotein and apolipoprotein profiles by nuclear magnetic resonance spectroscopy and targeted mass spectrometry measurements with PCSK9 levels in the community-based Bruneck study (n=656) revealed a positive association of plasma PCSK9 with small HDL, alongside a highly significant positive correlation between plasma levels of PCSK9 and apolipoprotein C3 (apoC3), an inhibitor of lipoprotein lipase. The latter association was replicated in an independent cohort, the SAPHIR study (Salzburg Atherosclerosis Prevention Program in Subjects at High Individual Risk; n=270). Thus, PCSK9-HDL association was determined during the postprandial response in two dietary studies (n=20 participants each, 8 time points). Peak triglyceride levels coincided with an attenuation of the PCSK9-HDL association, a loss of apoC3 from HDL, and lower levels of small HDL as measured by nuclear magnetic resonance spectroscopy. Crosslinking mass spectrometry upon isolated HDL identified PCSK9 as a potential HDL-binding partner. PCSK9 association with HDL was confirmed through size-exclusion chromatography and immuno-isolation. Quantitative proteomics upon HDL isolated from patients with coronary artery disease (n=172) returned PCSK9 as a core member of the HDL proteome. Combined interrogation of the HDL proteome and lipidome revealed a distinct cluster of PCSK9, phospholipid transfer protein, clusterin, and apolipoprotein E within the HDL proteome that was altered by sex and positively correlated with sphingomyelin content. Mechanistically, HDL facilitated PCSK9-mediated low-density lipoprotein receptor degradation and reduced low-density lipoprotein uptake through the modulation of PCSK9 internalization and multimerization.

**Conclusions:**

This study reports HDL as a binder of PCSK9 and regulator of its function. The combination of -omic technologies revealed postprandial lipemia as a driver of PCSK9 and apoC3 release from HDL.

**Registration:**

URL: https://www.clinicaltrials.gov; Unique identifier: NCT03191513; URL: https://www.isrctn.com; Unique identifier: ISRCTN20774126.

## Abstract 2

Supplemental Digital Content is available in the text.

**Meet the First Author, see p 973**

We have previously conducted apolipoprotein profiling by mass spectrometry (MS) in fasted plasma of the community-based Bruneck cohort, demonstrating stronger associations of apolipoproteins on TRL (triglyceride-rich lipoproteins) with incidences of atherosclerotic cardiovascular disease (CVD) than apoB.^1^ Notably, the Reduction of Cardiovascular Events with Icosapent Ethyl-Intervention Trial (REDUCE-IT) trial has since shown that triglycerides are indeed an important treatment target for reducing CVD in patients with elevated triglyceride levels despite the use of statins.^2^ In addition, we made a surprising observation that circulating PCSK9 (proprotein convertase subtilisin/kexin type 9) in humans is predominantly bound to HDL (high-density lipoprotein) and not to LDL (low-density lipoprotein)^3^ or Lp(a) (lipoprotein [a])^4^ as previously believed.^5^ The ability of PCSK9 to reside on multiple lipoprotein species is currently an understudied mode of PCSK9 regulation but could link PCSK9 activity to circulating lipid levels. LDL is thought to competitively inhibit the action of PCSK9 upon the LDLR (LDL receptor); however, whether HDL alters PCSK9 function remains unknown.^3^

Key questions that remain to be addressed are how PCSK9-lipoprotein interactions differ in plasma during the fasted and postprandial state as well as in patients with hyperlipidemia and coronary artery disease (CAD). In the present study, we investigated the associations between apolipoproteins and PCSK9 in fasted plasma from two prospective, community-based studies. The HDL composition was then interrogated by MS in healthy volunteers during postprandial lipemia. Through the use of crosslinking MS (XLMS), we identified PCSK9 as a binding partner of apoA1. We then analyzed the variations of the HDL proteome and lipidome in a cohort of patients with CAD. Finally, we demonstrate that similar to LDL, the interaction between HDL and PCSK9 alters PCSK9 functionality.

## Methods

An expanded Methods section is available in the Data Supplement.

### Data Availability

The data that support the findings of this study are available from the corresponding author upon reasonable request.

### Community-Based Studies

Nuclear magnetic resonance (NMR) spectroscopy–based lipoprotein profiling, PCSK9 measurements (DY3888, R&D Systems) and targeted apolipoprotein measurements by MS were conducted in the Bruneck Study. The Bruneck Study is a prospective, community-based survey of the epidemiology and pathogenesis of atherosclerosis and CVD. An age- and sex-stratified random sample of all inhabitants of Bruneck, Italy, all of White descent, was enrolled in 1990. In 2000, 702 subjects participated in the second quinquennial follow-up. PCSK9 measurements and targeted apolipoprotein measurements were repeated in the SAPHIR Study (Salzburg Atherosclerosis Prevention Program in Subjects at High Individual Risk). SAPHIR is a prospective cohort study conducted in 1770 healthy unrelated subjects (663 females and 1107 males aged 39 to 67 years) who were recruited by health screening programs in large companies in and around the city of Salzburg, Austria. For the current analysis, we used a nested case-control design. We selected all participants with incident primary CVD events (defined as myocardial infarction, ischemic stroke, or vascular death), all participants with CVD other than stroke and myocardial infarction, plus an age- and sex-matched subcohort (n=270).

### Postprandial Studies

Postprandial samples were analyzed from a double-blinded, 3-armed, randomized controlled trial (approved by King’s College London Research Ethics Committee, HR-16/17-4397) in healthy adults (n=20; 10 men, 10 women) aged 58 (SD±6.4) years. Samples were selected following consumption of the control test meal only containing 50 g rapeseed oil (61% 18:1n-9cis; 19% 18:2n-6cis) fed in the form of a muffin and a milkshake (to deliver 897 kcal, 50 g fat, 18 g protein, 88 g carbohydrate), following an overnight fast. Plasma samples were collected at hourly intervals 0-8 hours postprandially. A second, postprandial validation cohort in healthy subjects was assessed (n=20, 8 time points), adhering to the same study design and test meal outlined above. Ethical approval for the study was obtained from the relevant research ethics committees in the United Kingdom (NREC 08/H1101/122) and the Netherlands (MEC 09-3-009), and written informed consent was given by participants. NMR-based lipoprotein profiling and PCSK9 measurements (DY3888, R&D Systems) were conducted across all samples. HDL immuno-isolation coupled with label-free quantitative proteomics was conducted upon a subset of plasma samples.

### Crosslinking MS

Protein-protein interactions within pooled plasma, HDL isolated by immuno-isolation (Genway Biotech), and HDL isolated by ultracentrifugation from healthy donors were interrogated using the MS-compatible crosslinker disuccinimidyl sulfoxide (Thermo Fisher). Crosslinked samples underwent in-solution digestion before carbon 18 (C18) purification and strong cation exchange fractionation. Crosslinked peptides were analyzed using an Orbitrap mass analyzer (Orbitrap Fusion Lumos, Thermo Scientific). MS data were searched within Proteome Discoverer (Thermo Scientific) using the in-built XlinkX node.^6^

### Assessment of PCSK9-HDL Association

The large-scale depletion and isolation of HDL from pooled plasma from healthy volunteers was achieved through the conjugation of anti-human apoA1 antibody (Academy Bio-Medical) with cyanogen bromide–activated sepharose. High-performance size-exclusion chromatography (SEC, Superose 6 Increase 10/300 GL column) was conducted upon healthy pooled volunteer plasma and PCSK9 preincubated with commercially sourced lipoproteins (Sigma). Lipoprotein-associated PCSK9 was also measured using an in-house ELISA, as previously described.^5^

### CAD Patient Cohort

From 2006 to 2007, blood samples were prospectively taken from consecutive patients ≥18 years of age presenting either with acute myocardial infarction (ST-segment–elevation or non–ST-segment–elevation myocardial infarction, as defined by the European Society of Cardiology guidelines), stable CAD, part of whom underwent percutaneous coronary intervention, or microvascular angina at the Clinic of Cardiology, West German Heart Center, University Hospital Essen, and the Alfred Krupp Hospital Essen. Patients with CAD who underwent percutaneous coronary intervention were symptomatic for the disease, typically manifesting as angina pectoris, but were ruled out of having myocardial infarction. Angiographic assessment identified a stenosis of at least 50% in at least one coronary artery in these patients. Microvascular angina was defined as symptomatic patients with coronary stenosis of <50% but with reduced coronary flow reserve as measured by intracoronary Doppler. The study was approved by the Local Ethics Committee (University Hospital Essen, 02-2965). HDL was isolated by potassium bromide (kBr)-sequential density gradient ultracentrifugation following an established protocol.^7^ Both label-free and tandem-mass tag (TMT)–based quantitative proteomics, as well as targeted lipidomics, was conducted upon isolated HDL samples.

### In Vitro Studies

The human hepatocellular carcinoma cell line (HepG2, ECACC 85011430) was used as an in vitro model of cellular cholesterol metabolism. Cells were treated with stated concentrations of recombinant HIS (polyhistidine)-tagged PCSK9 (ACRO Biosystems, PC9-H5223), rHDL (reconstituted HDL, Genway), and ucHDL (ultracentrifuge-isolated HDL, Merck). The cellular uptake of LDL was determined using the Image-iT LDL Uptake Kit, BODIPY-FL, according to the manufacturer’s instructions (Thermo Fisher).

### Statistical Analysis

Proteomic and lipidomic data were filtered to keep only molecules with <70% missing values. The remaining missing values were imputed using k-nearest neighbor-imputation method with k equal to 10. The quantities of molecules were scaled using log_2_ transformation. All statistical comparisons have been conducted using nonparametric tests unless otherwise stated. *P* values were adjusted using Benjamini Hochberg for multiple testing when appropriate, keeping proteins with a false discovery rate threshold of 5%.

## Results

### Lipoprotein Profiling Identifies PCSK9 Association With Small HDL

**Figure 1.**
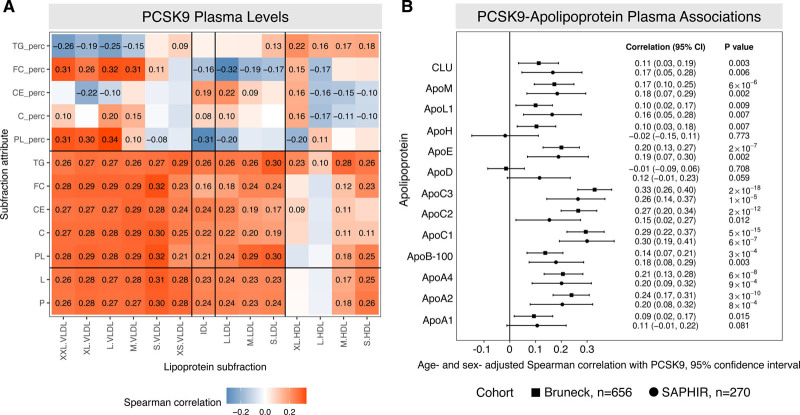
**Integrated lipoprotein analysis in plasma.** **A**, Nuclear magnetic resonance (NMR) lipoprotein analysis and targeted apolipoprotein profiling was conducted in the Bruneck study (n=656). Plasma PCSK9 (proprotein convertase subtilisin/kexin type 9) levels, as measured by ELISA, were correlated against NMR lipoprotein attributes including; particle number (P), lipid contents (L), phospholipids (PL), total cholesterol (C), cholesterol esters (CE), free cholesterol (FC), triglycerides (TG), and lastly, each lipid class is also represented as a percentage of total lipids (perc). The lipoprotein particles are resolved by size: extremely large (XXL); extra large (XL); large (L); medium (M); small (S); and very small (XS). Only correlations with a *P*<0.05 are represented. Each Spearman coefficient is displayed. **B**, Plasma PCSK9 levels were correlated with the apolipoprotein profiles as measured by targeted mass spectrometry with authentic heavy standards in both the Bruneck (n=656) and SAPHIR (Salzburg Atherosclerosis Prevention Program in Subjects at High Individual Risk; n=270) cohorts. Note the strong association of PCSK9 plasma levels with C apolipoproteins. *P* values were not adjusted for multiple testing. Overall, 26 tests were performed, for 13 apolipoproteins in each of the 2 cohorts. The Bonferroni adjusted threshold of significance (at the 0.05 level) is 0.0019. CIs are bootstrap percentile CIs based on 1000 bootstrap resamples. CLU indicates clusterin (apolipoprotein J); HDL, high-density lipoprotein; LDL, low-density lipoprotein; and VLDL, very low-density lipoprotein.

NMR analysis and PCSK9 measurements were conducted in fasted plasma samples from the community-based Bruneck cohort (n=656; Figure 1A, Table I in the Data Supplement). As expected, PCSK9 positively correlated with the particle number and lipid content of the main circulating apoB-containing particles, VLDL (very low-density lipoprotein), IDL (intermediate-density lipoprotein), and LDL, but this correlation was only of moderate strength. Remarkably, PCSK9 returned a similar positive correlation with the particle number of S.HDL (small HDL, r=0.26, *P*=2.1×10^-11^, Figure 1A). For validation, measurements of PCSK9 were performed in isolated small, dense HDL (HDL3) and larger, less dense HDL (HDL2), confirming an enrichment of PCSK9 within HDL3 (Figure I in the Data Supplement). Besides the PCSK9-S.HDL correlation, a differential association between PCSK9 and lipoprotein triglyceride percentage content was observed in larger VLDL (inverse association: medium VLDL, large VLDL, extra large, extremely large VLDL) and all HDL particles (positive association, Figure 1A).

To further support an effect of PCSK9 on triglyceride metabolism, PCSK9 measurements were correlated with plasma apolipoprotein measurements (Figure 1B).^1^ Among the 13 apolipoproteins measured by targeted MS, PCSK9 plasma levels showed the strongest correlation with C apolipoproteins, in particular with apoC3, an inhibitor of LPL (lipoprotein lipase), the enzyme primarily responsible for the hydrolysis of plasma triglycerides.^8^ The strong positive associations between plasma levels of PCSK9 and C apolipoproteins were replicated in an independent community-based cohort, the SAPHIR study (n=270, Figure 1B).

### PCSK9 Kinetics During Postprandial Lipemia

**Figure 2.**
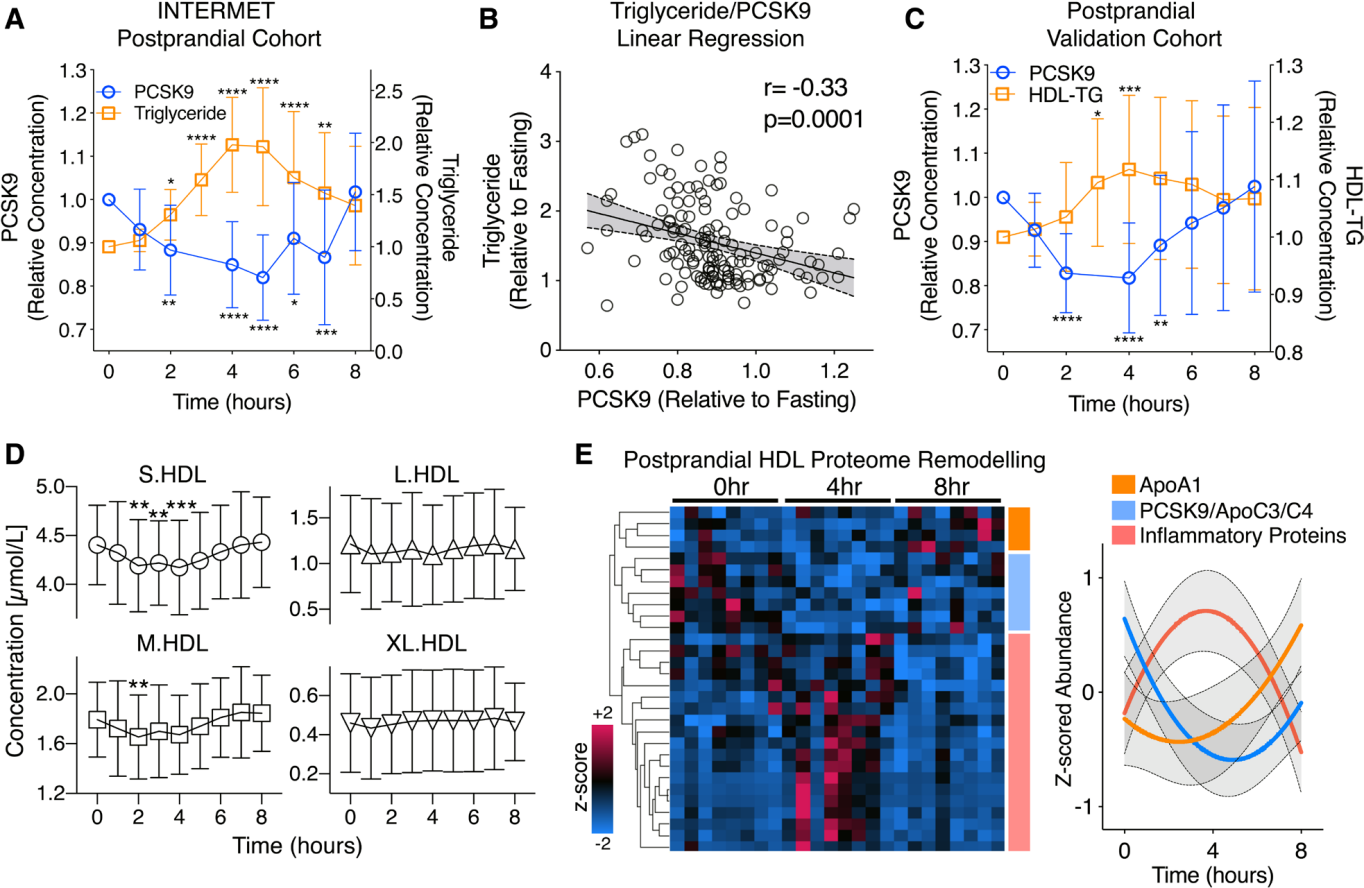
**HDL (high-density lipoprotein) composition during the postprandial response.** **A**, Postprandial plasma samples were obtained from 20 healthy individuals at 8, hourly time points (INTERMET [acute effects of interesterification of commercially used fats on postprandial fat metabolism] study). Plasma PCSK9 (proprotein convertase subtilisin/kexin type 9) levels were measured, alongside triglycerides (TGs). **B**, A linear regression analysis between the PCSK9 and TG postprandial response is represented. **C**, Postprandial plasma samples from a further 20 healthy individuals at 8, hourly time points (validation cohort) were assessed for PCSK9 and HDL-TG content. **D**, Nuclear magnetic resonance–based lipoprotein analysis was conducted over the postprandial time course, and the particle concentration of S.HDL (small HDL), M.HDL (medium HDL), L.HDL (large HDL), and XL.HDL (extra-large HDL) is shown. **E**, Label-free proteomics was conducted upon HDL immuno-isolated from postprandial plasma samples (n=8, 3 time points), and significantly changing proteins over 8 h are represented as a heat map and protein clusters are shown graphically. Significance was determined using the nonparametric Friedman test with Dunn correction, *P<0.05, **P<0.005, ***P<0.0005, ****P<0.0001.

To interrogate the interaction between PCSK9 and triglycerides, we assessed the postprandial response in healthy volunteers. In the INTERMET (acute effects of interesterification of commercially used fats on postprandial fat metabolism) postprandial cohort (n=20, 8 time points, Table II in the Data Supplement), the test meal contained 50 g fat and 85 g carbohydrate (850 kcal, 15 g protein). PCSK9 measurements by ELISA confirmed a reduction of circulating PCSK9 levels compared with the fasted state (Figure 2A). This reduction coincided with peak postprandial lipemia, within the first 5 hours. Circulating PCSK9 and triglyceride levels reverted back to baseline concentrations at 8 hours postprandially (Figure 2A). A significant, negative correlation was observed between the postprandial PCSK9 and triglyceride responses (r=−0.33, *P*=0.0001, Figure 2B). The plasma reduction of PCSK9 was replicated in a second postprandial cohort, adhering to the same test meal, with an observed triglyceride peak at 4 hours, 50% greater than baseline (8 time points, n=20, Figure 2C).^9^ Additionally, NMR-based lipoprotein analyses in the postprandial validation cohort revealed triglyceride loading of HDL (Figure 2C) as well as a significant reduction in the particle concentrations of S.HDL and M.HDL between 2 and 4 hours when lower PCSK9 levels were observed (Figure 2D). In contrast, L.HDL (large HDL) and XL.HDL (extra-large HDL) levels remained stable (Figure 2D).

### Postprandial HDL Proteome Remodeling

To further investigate the interaction of PCSK9 with the apoC family of apolipoproteins, we studied postprandial HDL remodeling by proteomics. Ultracentrifugation is known to strip proteins, including PCSK9,^10,11^ from lipoproteins during isolation. HDL was, therefore, immuno-isolated over the postprandial time course at 0, 4, and 8 hours (n=8 each) and analyzed by label-free MS. One hundred sixty-three proteins were identified in this analysis. Consistent with previous results,^12^ apoA1 content of HDL was greatest at 8 h postprandially (orange cluster, 4 proteins, Figure 2E). In contrast, a cluster of inflammatory proteins increased in abundance upon HDL at 4 hours postprandially, before returning to fasted levels (red cluster, 19 proteins, Figure 2E). The postprandial reduction in HDL-bound PCSK9 as confirmed by proteomics was accompanied by a reduction of C apolipoproteins, including apoC3 (blue cluster, 8 proteins, Figure 2E, Figure IIA in the Data Supplement). Details on HDL proteome remodeling over the postprandial time period are represented in Table III in the Data Supplement. The postprandial difference in HDL-bound PCSK9 was further validated using an ELISA and mirrored the reduction observed in the circulation (Figure IIB in the Data Supplement).

### XLMS-Based Interrogation of the Plasma and HDL Interactome

**Figure 3.**
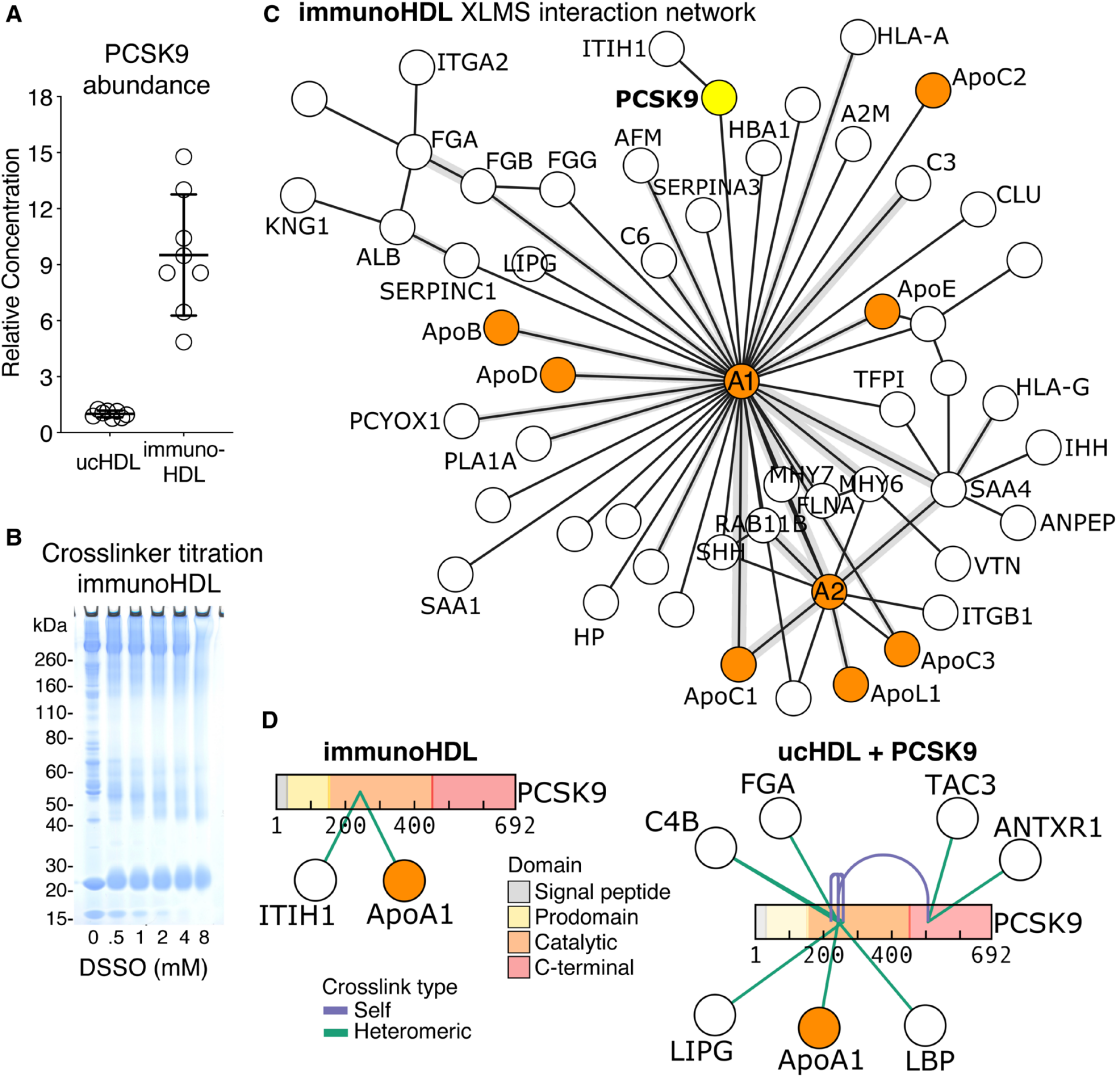
**Crosslinking mass spectrometry (XLMS) identifies PCSK9 (proprotein convertase subtilisin/kexin type 9) as a potential HDL (high-density lipoprotein) interaction partner.** **A**, Ten micrograms of HDL protein (n=8) isolated by either ultracentrifugation (uc HDL) or immunocapture (immunoHDL) were directly compared for PCSK9 content by ELISA. **B**, Titration of the MS-cleavable crosslinker disuccinimidyl sulfoxide (DSSO) was conducted to determine optimal DSSO concentration, resulting in 1 mmol/L to be used for subsequent analyses. **C**, ImmunoHDL (n=8 healthy subjects, pooled) was analyzed by XLMS and identified interprotein crosslinks are represented as an interaction network. Apolipoproteins are highlighted in orange. PCSK9 is highlighted in yellow. Crosslink density is represented as gray area. **D**, Recombinant PCSK9 was spiked into ucHDL before XLMS analysis. PCSK9 crosslinks identified are represented for both immunoHDL and spiked ucHDL. XLMS data visualization was conducted using xiview.org. ANTXR1 indicates anthrax toxin receptor 1; C4B, complement factor 4B; FGA, fibrinogen alpha chain; ITIH1, interalpha-trypsin inhibitor heavy chain 1; LBP, lipopolysaccharide-binding protein; LIPG, endothelial lipase; and TAC3, tachykinin-3.

HDL has the most complex proteome among lipoproteins, yet protein interactions on HDL have not been comprehensively explored. Thus, we employed XLMS to probe protein-protein interaction networks on HDL at a proteome-wide scale. XLMS utilizes physical proximity constraints in the study of protein-protein interactions. HDL isolated by immunocapture (immunoHDL) had 10× more PCSK9 than HDL isolated by ultracentrifugation (ucHDL) per µg of HDL protein, as measured by ELISA (Figure 3A). Due to this apparent protein loss from HDL the initial XLMS experiments were performed on immunoHDL.

To study protein-protein interaction networks, the MS-cleavable crosslinker disuccinimidyl sulfoxide was used. Our XLMS workflow coupled with strong cation exchange peptide fractionation was first developed using BSA (Figure III in the Data Supplement). Pooled plasma and immunoHDL from healthy volunteers were used for XLMS. XLMS conducted upon plasma enabled the identification of 128 crosslinks, of which 28 were interprotein crosslinks and are represented as an interaction network (Figure IV in the Data Supplement). Only protein interactions among the most abundant apolipoproteins on HDL (apoA1-apoA2) could be captured at a plasma level using XLMS. In comparison, XLMS of immunoHDL identified 857 crosslinks, of which 141 were interprotein links. The two core interaction hubs identified were apoA1 and apoA2 forming distinct protein interaction networks (Figure 3C). PCSK9 was revealed to interact with apoA1 and the hyaluronan binder ITIH1 (interalpha-trypsin inhibitor heavy chain 1) in immunoHDL (Figure 3C).

To further probe interaction partners of PCSK9 within HDL, ucHDL was spiked with recombinant PCSK9 before XLMS analysis to compensate for protein loss during isolation. The interaction between PCSK9 and apoA1 was replicated (Figure 3D). Interestingly, the main crosslinked lysine residue within the catalytic domain of PCSK9, Lys243, is within the region of PCSK9 responsible for binding to the EGF (epidermal growth factor)-A domain of the LDLR.^13^ Moreover, 2 protein interaction hubs within PCSK9 were identified: other potential interactors of spiked PCSK9 with ucHDL included LIPG (endothelial lipase), LBP (lipopolysaccharide-binding protein), C4B (complement factor 4B), and FGA (fibrinogen), as well as TAC3 (tachykinin-3) and ANTXR1 (anthrax toxin receptor 1; Figure 3D).

### Confirmation of PCSK9-HDL Association

**Figure 4.**
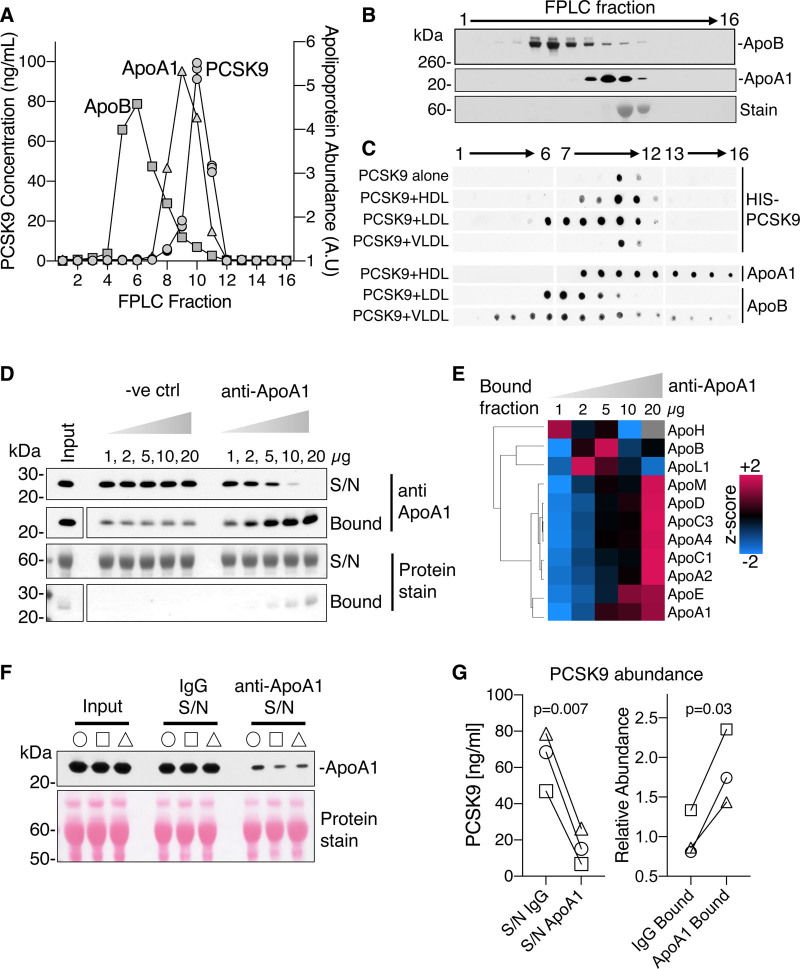
**Confirmation of PCSK9 (proprotein convertase subtilisin/kexin type 9)-HDL (high-density lipoprotein) interaction.** **A**, Pooled human plasma from healthy subjects (n=3) was separated by size-exclusion chromatography (SEC) and PCSK9 concentrations in collected fractions were determined by ELISA. **B**, Apolipoprotein abundances in SEC fractions as determined by immunoblotting. **C**, Recombinant HIS (polyhistidine)-tagged PCSK9 (20 µg/mL) was incubated with ucHDL (ultracentrifuge-isolated HDL), LDL (low-density lipoprotein), and VLDL (very low-density lipoprotein) before SEC separation; PCSK9 alone served as control. **D**, An anti–apoA1 immunoprecipitation method from human plasma was validated by immunoblotting. **E**, Mass spectrometry analysis of the apolipoproteins in the bound fraction of the anti–apoA1 pull downs are represented as a heat map. **F**, Plasma samples (10 µL, n=3 healthy volunteers) were depleted of apoA1. **G**, PCSK9 abundances were measured by ELISA in apoA1 depleted plasma (supernatant, S/N) and in the bound fractions. Significance was determined by a paired-Student *t* test. A.U indicates arbitrary units; FPLC, fast protein liquid chromatography; and ve ctrl, isotype control.

To confirm the interaction between apoA1 and PCSK9, pooled plasma from healthy volunteers was separated by SEC. The majority of PCSK9 coeluted with total protein and apoA1, rather than apoB (Figure 4A and 4B). In addition, recombinant HIS-tagged PCSK9 was incubated with either HDL, LDL, or VLDL isolated by ultracentrifugation before SEC separation. Prior incubation of PCSK9 with HDL and LDL led to a shift in the PCSK9 elution profile when compared with PCSK9 alone and PCSK9 with VLDL (Figure 4C). Furthermore, utilizing a different SEC column, the shift in PCSK9 elution as a consequence of preincubation with HDL was confirmed (Figure V in the Data Supplement). The purity of recombinant PCSK9 and lipoproteins utilized in this experiment was confirmed by MS (Figure VI in the Data Supplement).

Next, an anti–apoA1 immuno-depletion method was developed. Initial antibody validation highlighted an efficient removal and enrichment of apoA1, paralleled by an enrichment of HDL-associated apolipoproteins (Figure 4D and 4E). Apart from apoM and apoA4, all of these apolipoproteins were returned as interaction partners by XLMS in immunoHDL (Figure 3C). To remove a large amount of HDL from plasma, anti–apoA1 antibody was conjugated to sepharose, whereas IgG-conjugated sepharose was used as control to account for nonspecific interactions. The removal of apoA1 from plasma was validated by immunoblotting (n=3, Figure 4F). The immuno-depletion of HDL from plasma led to a greater reduction of PCSK9 levels as measured by ELISA when compared with IgG control (n=3, Figure 4G, left). This result was mirrored in the greater enrichment of PCSK9 within the anti–apoA1 bound fraction (n=3, Figure 4G, right).

### PCSK9 Is a Core Member of the HDL Proteome

**Figure 5.**
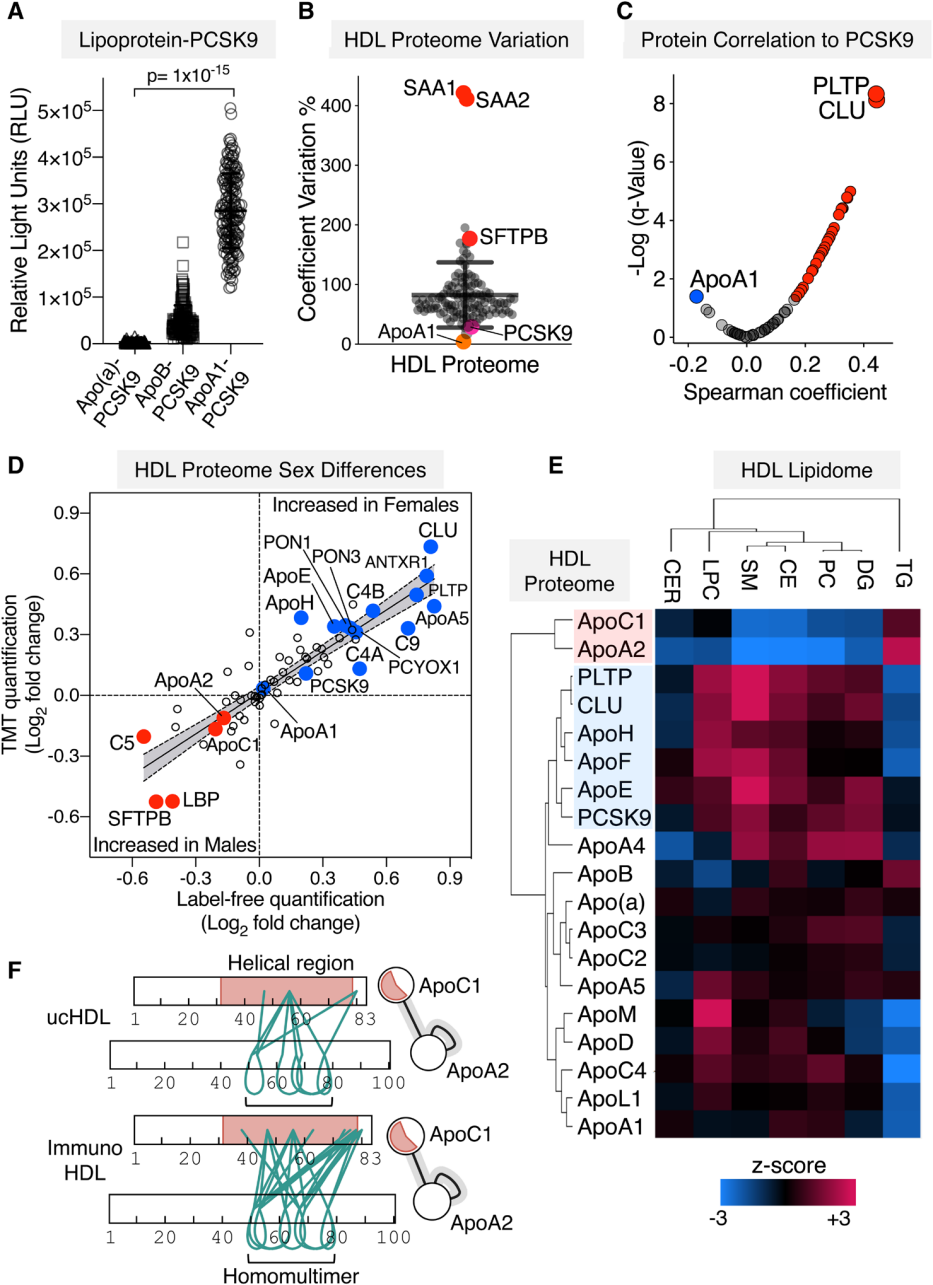
**Integrated proteomics and lipidomics analysis of HDL (high-density lipoprotein) in patients with coronary artery disease (CAD).** **A**, PCSK9 (proprotein convertase subtilisin/kexin type 9) distribution across lipoprotein fractions within a cohort of 172 patients with varying CAD-related phenotypes was assessed using a modified sandwich ELISA. Significance was determined using the Kruskal-Wallis test across the groups. **B**, ucHDL (ultracentrifuge-isolated HDL) from these patients with CAD (n=191, including samples from patients with 6 mo follow-up) was analyzed by quantitative proteomics. The coefficients of variation in HDL protein abundances, as measured by label-free mass spectrometry (MS), were calculated across the whole cohort. **C**, PCSK9 protein correlations against the core HDL proteome are represented as a volcano plot. **D**, Quantitation by label-free and tandem-mass tag (TMT) proteomics upon ucHDL revealed proteins that were altered by sex (males, n=98. females n=66). Significant proteins in at least one method are labeled; fold changes across methodologies were compared using a linear regression analysis. **E**, Three hundred sixty-five lipid species were quantified in HDL by targeted MS with reference standards. A Spearman correlation matrix was generated between the sum of each lipid species in a respective class and the HDL apolipoprotein profile, as well as PCSK9, PLTP, and CLU. A hierarchical cluster analysis is represented as a heat map. **F**, Crosslinking mass spectrometry (XLMS) analysis of immunoHDL (immuno-isolated HDL) and ucHDL revealed a strong protein-protein interaction between apoA2 and apoC1, green lines represent crosslinks (xiview.org). AC indicates acylcarnitines; ANTXR1, anthrax toxin receptor 1; C4B, complement factor 4B; CE, cholesterol esters; CER, ceramides; CLU, clusterin; DG, diacylglycerides; LBP, lipopolysaccharide-binding protein; LPC, lyso-phosphatidylcholine; PC, phosphatidylcholine; PCYOX, prenylcysteine oxidase; PLTP, phospholipid transfer protein; PON, paraoxonase; RLU, relative light units; SAA, serum amyloid A; SFTPB, surfactant protein B; SM, sphingomyelins; and TG, triglycerides.

Besides its well-established role in reverse cholesterol transport, HDL is protein-rich, providing alternative explanations for its CVD-related pathological functions. To obtain comprehensive insights into the protein and lipid composition of HDL in patients with CAD and relate it to PCSK9, HDL was isolated from 172 patients with CAD. Their clinical characteristics are given in the Table. Plasma PCSK9 levels were highest in patients with stable CAD due to the known effect of statins on raising PCSK9 expression (Table and Figure VII in the Data Supplement).^14^ The lipoprotein distribution of PCSK9 was determined using a modified sandwich ELISA across the entire cohort.^15^ A greater signal for apoA1–PCSK9 was observed when compared to apoB-PCSK9 and apo(a)-PCSK9 (Figure 5A). The HDL proteome was then interrogated by quantitative proteomics and overlapped with those in the HDL proteome database (Figure VIII in the Data Supplement). To determine interprotein relationships, only proteins quantifiable in all HDL samples were retained. This HDL proteome of 66 proteins included PCSK9 and revealed distinct clusters of HDL-associated proteins (Figures IX and X in the Data Supplement).

**Table.**
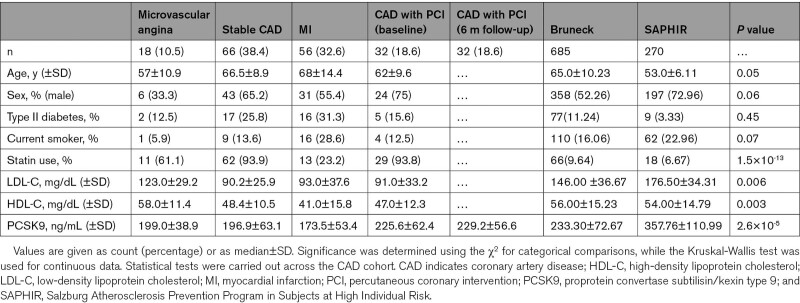
Clinical Characteristics of CAD Cohort With HDL Isolation (n=172) and Community-based Studies

Key drivers of the variation in the HDL proteome were inflammatory-related, such as acute-phase proteins SAA1 and SAA2 (serum amyloid A1 and A2) and the lung-derived pulmonary SFTPB (surfactant protein B); in contrast, PCSK9 levels were comparatively stable within the HDL proteome (Figure 5B). The strongest and most significant proteins to correlate to PCSK9 within the HDL proteome were PLTP (phospholipid transfer protein), as well as CLU (clusterin; Figure 5C). To validate the quantitative accuracy of the MS measurement, PCSK9 was measured by ELISA. Both measurements were highly correlated (*r*=0.76, n=165).

### Sex Differences in the PCSK9 Association With HDL

Circulating PCSK9 levels are known to be influenced by sex.^16^ Next, we int

errogated quantitative protein differences in the HDL proteome by tandem-mass tag-multiplexing (Table IV in the Data Supplement) to complement label-free quantitation (Figure 5D, Table V in the Data Supplement). Linear regression analyses conducted upon the HDL proteome of the CAD cohort returned sex to be the only clinical variable statistically associated with HDL-PCSK9 levels (coefficient=0.44, *P*=0.02). PCSK9 was significantly enriched in HDL isolated from females, alongside PLTP, CLU, and apoE (Figure 5D).

ApoA2, apoC1 and SFTPB were higher in the HDL of male patients (Figure 5D). The enrichment of SFTPB within the HDL proteome of males was confounded by the increased prevalence of smokers (Figure XI in the Data Supplement). Interestingly, smoking status was revealed to be responsible for enrichment of other lung-derived proteins in the HDL proteome including SCGB3A1 and A2 (secretoglobin family 3A member 1 and 2; Figure XI in the Data Supplement).

Due to the strength of association between PCSK9 and PLTP, a core regulator of HDL phospholipid content, targeted MS-quantitation of 365 lipid species was conducted in HDL from the CAD cohort. A hierarchical cluster analysis performed upon correlation matrices between the protein profile and lipidome of HDL not only replicated the distinct relationship between PCSK9, PLTP, and CLU (Figure 5C) but also included apoE when the HDL lipidome is included (Figure 5E). The PCSK9-associated protein cluster revealed the strongest positive correlation with the SM (sphingomyelin) content of HDL (r=0.33, *P*=0.001) but was inversely associated with triglycerides (Figure 5E). Instead, apo apoA2 and apoC1 were positively associated with triglyceride levels within HDL (r=0.28, *P*=0.0004, Figure 5E). Further interrogation of the XLMS datasets revealed a large number of high-confidence crosslinks between apoA2 and apoC1 in both immunoHDL and ucHDL (Figure 5F), confirming that this cluster represented a strong protein interaction that withstands ultracentrifugation.

### HDL Facilitates PCSK9-Mediated LDLR Degradation

**Figure 6.**
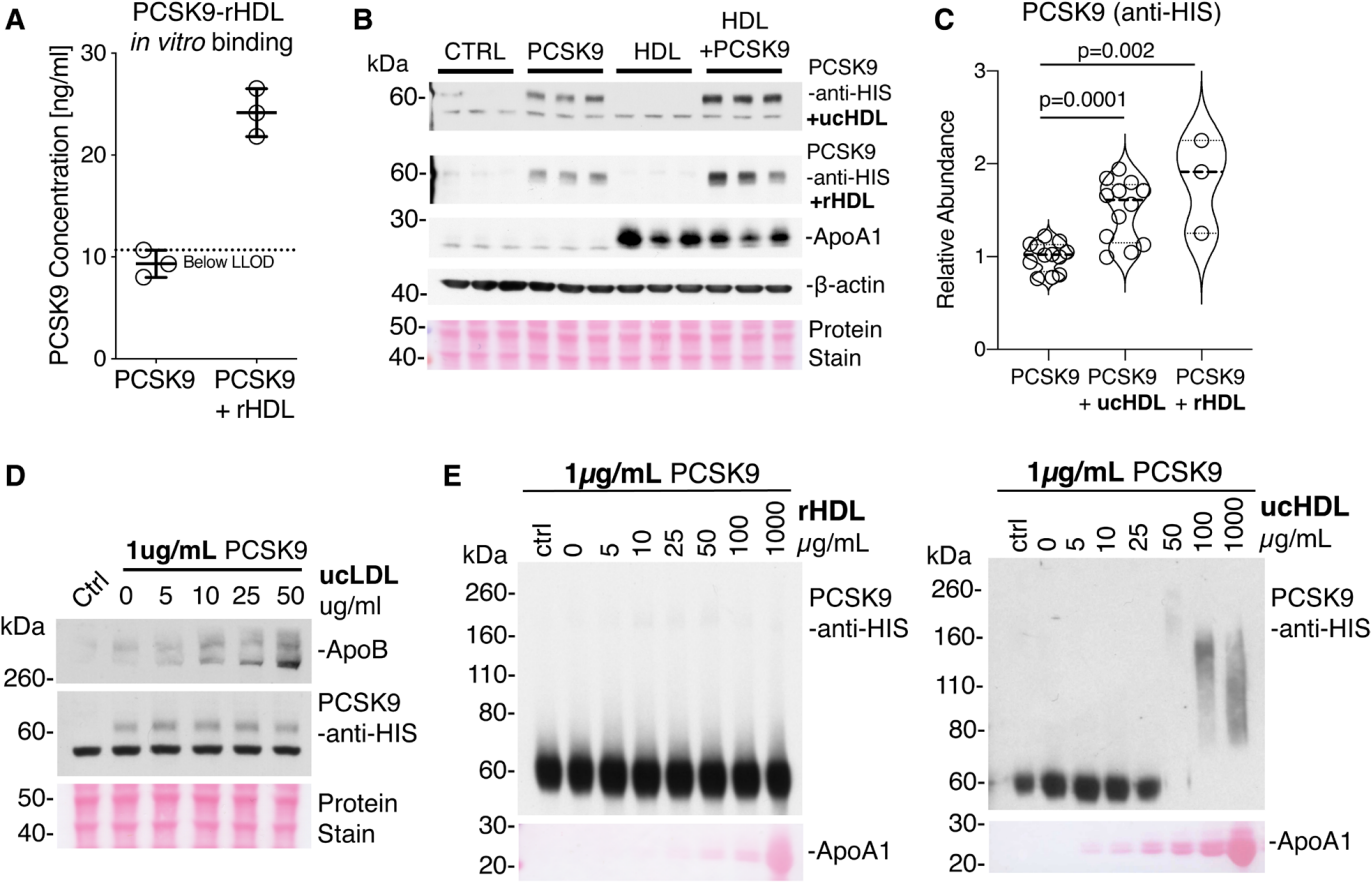
**HDL (high-density lipoprotein) potentiates PCSK9 (proprotein convertase subtilisin/kexin type 9) uptake and multimerization.** **A**, PCSK9 and rHDL (reconstituted HDL) were coincubated before HDL immuno-isolation to demonstrate the interaction between rHDL and PCSK9. PCSK9 alone was run through the HDL immunoisolation column as a negative control. **B**, HepG2 cells were treated with HIS (polyhistidine)-tagged PCSK9 (5 µg/mL), rHDL, or ucHDL (ultracentrifuge-isolated HDL; 25 µg/mL) or a combination of rHDL or ucHDL and HIS-tagged PCSK9 for 6 h before immunoblot analysis for PCSK9 cellular uptake. **C**, Densitometry analysis of 3 independent replicates. Significance was determined using an independent *t* test. **D**, HepG2 cells were treated with HIS-tagged PCSK9 (1 µg/mL), in the presence of increasing amounts of ucLDL (ultracentrifuge-isolated low-density lipoprotein; 0–50 µg/mL). The control lane (Ctrl) represents PCSK9 only. **E**, Recombinant HIS-tagged PCSK9 at a concentration of 1 µg/mL was incubated in the presence of an increasing concentration of rHDL or ucHDL. Immunoblot analysis was then conducted, with equal amounts of PCSK9 loading for each sample. The control lane represent PCSK9 alone incubated at 4°C. Total protein stain is used to visualize apoA1. LLOD indicates lower limit of detection.

Lastly, we determined whether HDL alters PCSK9 function. Commercially sourced rHDL was prepared from apoA1 and phosphatidylcholine. Consistent with our XLMS results (Figure 3C and 3D), recombinant PCSK9 was capable of associating with rHDL in vitro (Figure 6A). Treatment of HepG2 cells with recombinant PCSK9 and either rHDL or ucHDL increased PCSK9 uptake compared with treatment with PCSK9 alone (Figure 6B and 6C). PCSK9 uptake was not influenced by LDL (Figure 6D). In contrast, ucHDL but not rHDL was able to promote multimerization of PCSK9 (1 µg/mL) at physiological concentrations (Figure 6E). The promotion of PCSK9 multimerization also occurred at higher PCSK9 concentration (10 µg/mL, Figure XII in the Data Supplement), whereas ucLDL (ultracentrifuge-isolated LDL) induced a differing PCSK9 multimeric shift compared with ucHDL at equivalent concentrations (Figure XII in the Data Supplement).

**Figure 7.**
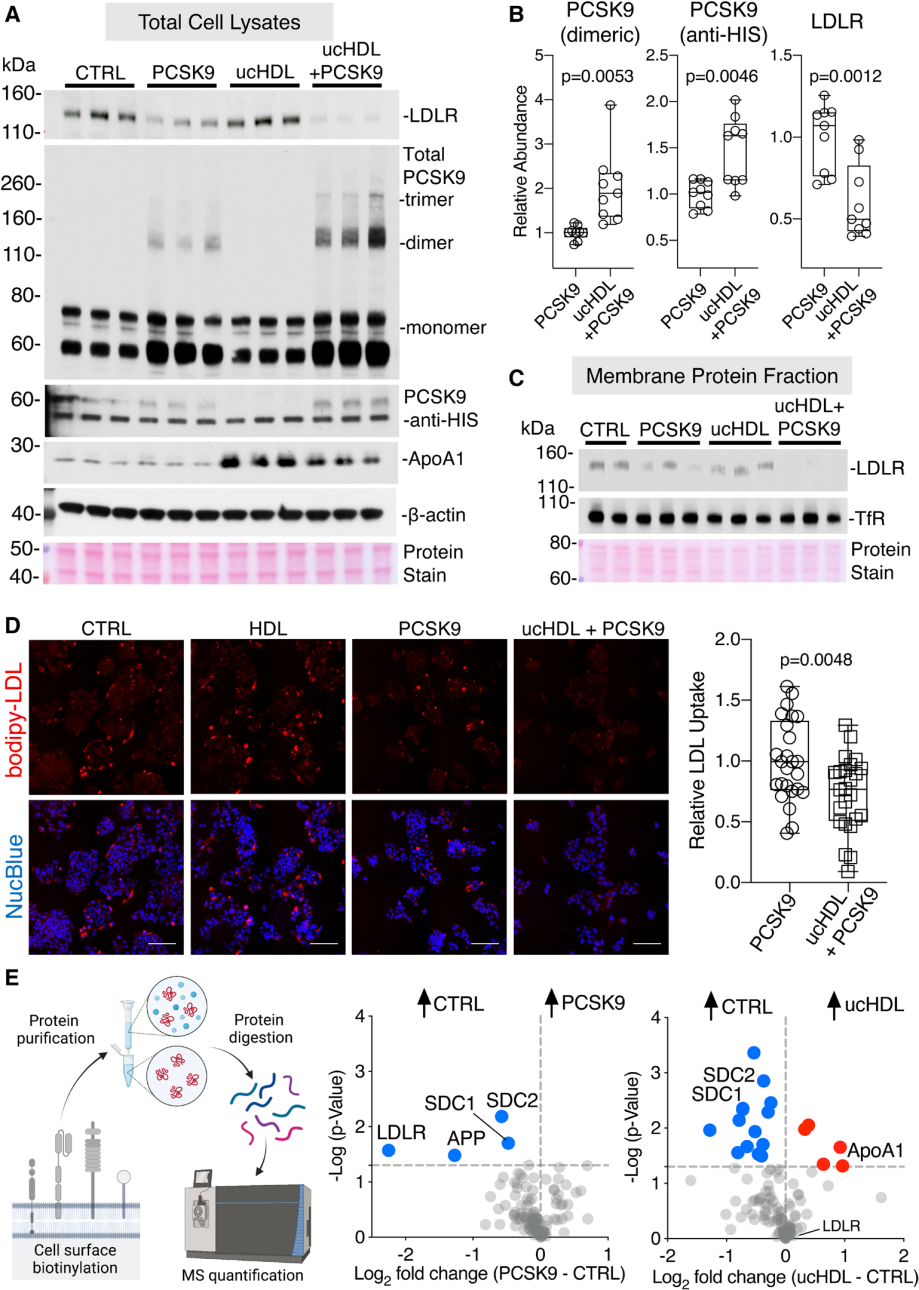
**HDL (high-density lipoprotein) facilitates PCSK9 (proprotein convertase subtilisin/kexin type 9)-mediated LDLR (low-density lipoprotein receptor) degradation.** **A**, HepG2 cells were treated with HIS (polyhistidine)-tagged PCSK9 (1 µg/mL), ucHDL (ultracentrifuge-isolated HDL; 50 µg/mL), or a combination of ucHDL and HIS-tagged PCSK9 for 6 h in the presence of actinomycin D (5 µg/mL), before immunoblot analysis. **B**, Densitometry analysis of 3 independent replicates. **C**, The same coincubation experiment was repeated by isolating the membrane protein fraction through cell surface biotinylation and NeutrAvidin agarose enrichment. **D**, HepG2 cells were treated with HIS-tagged PCSK9 (1 µg/mL), ucHDL (50 µg/mL), or a combination of ucHDL and HIS-tagged PCSK9 for 6 h in the presence of actinomycin D, before the addition of bodipy-LDL (12 µg/mL) for 3 h. Representative fluorescent images for each condition are represented, LDL uptake can be seen in red and a nuclear counterstain in blue (NucBlue), scale bar represents 100 µm. Quantitative analysis of 10 cellular areas for each condition was conducted in ImageJ, summed particle intensities per area were taken, normalized to cell nuclei count. Data is representative of 3 independent experiments. **E**, Cell surface proteins were isolated from HepG2 cells treated with HIS-tagged PCSK9 (1 µg/mL) or ucHDL (50 µg/mL) for 6 h in the presence of actinomycin D before label-free quantification by mass spectrometry (MS). Proteins with cell surface localization were retained and changes across conditions are represented in volcano plots. Significance was determined using a *t* test with Welch correction. APP indicates amyloid precursor protein; CTRL, control; SDC, syndecan; and TfR, transferrin receptor.

Treatment of HepG2 cells with PCSK9 and ucHDL reduced cellular LDLR protein levels to a greater extent as compared to PCSK9 alone (Figure 7A and 7B). Findings remained consistent when larger amounts of ucHDL were used (Figure XIIIA in the Data Supplement). This coincided with higher uptake and increased multimerization of PCSK9 (Figure 7A and 7B). Both total and cell membrane LDLR levels were reduced upon incubation of cells with PCSK9 and ucHDL (Figure 7C). Actinomycin D was used to prevent the compensatory increase in LDLR upon treatment with ucHDL or rHDL (Figure XIV in the Data Supplement). Even without actinomycin D, the PCSK9 response was maintained (Figure XIIIB in the Data Supplement). The functional consequence of reduced LDLR is an inability to internalize LDL. HepG2 cells pretreated with PCSK9 and ucHDL had reduced uptake of fluorescent LDL in comparison to cells treated with PCSK9 alone (Figure 7D).

Finally, to determine potential LDLR-independent mechanisms of PCSK9 uptake, cell surface proteins were isolated and analyzed by label-free MS. As expected, a robust reduction in the LDLR upon PCSK9 treatment was observed (Figure 7E). Interestingly, membrane abundances of both SDC1 (syndecan 1) and SDC2 (syndecan 2) were equally reduced by ucHDL and PCSK9 treatments (Figure 7E, Table VI in the Data Supplement). SDC1 and SDC2 carry heparan sulfate and chondroitin sulfate chains, and binding to proteoglycans constitutes a common mechanism that facilitates cellular uptake of PCSK9 and HDL.

## Discussion

The present study includes -omics results from 5 clinical cohorts: PCSK9 and apolipoprotein measurements by targeted MS in two community-based studies; postprandial data from two double-blinded, randomized controlled trial dietary studies and MS-based proteomics and lipidomics in HDL isolated from patients with CAD. Lastly, HDL was shown to act as a facilitator of PCSK9-driven LDLR degradation. Thus, the interaction between HDL and PCSK9 is a previously unrecognized mechanism by which lipoproteins may contribute to the regulation of PCSK9 activity.

### PCSK9, ApoC3, and Triglycerides

A key rationale to study the different cohorts was the well-known inverse correlation of HDL and triglycerides. Analysis of fasted plasma in the prospective, community-based Bruneck and SAPHIR cohorts revealed a significant positive association between PCSK9 and apoC3, consistent with a link between PCSK9 and the regulation of TRL metabolism. ApoC3 interferes with apoC2–mediated activation of LPL and thereby raises plasma triglycerides.^17^ Additionally, apoC3 regulates TRL metabolism by an LPL-independent pathway.^18,19^ The Vaisar lab recently described that HDL triglycerides increase postprandially, which was paralleled by an enrichment of apoA1, features of HDL remodeling that we also observe.^10^ Of the limited human postprandial studies to date that have investigated the PCSK9 response,^20,21^ none have addressed PCSK9-lipoprotein binding. Our study is the first to reveal PCSK9 on HDL to be significantly reduced postprandially. Alongside PCSK9, the apoC3 content on HDL decreased postprandially, corroborating the positive association we observed between PCSK9 and apoC3 in the Bruneck and SAPHIR cohorts.

### PCSK9 During Postprandial HDL Remodeling

Apolipoproteins share common amphipathic helices that enable their exchange across lipoprotein species. A transient amphipathic helix within the N-terminal region of PCSK9 is responsible for its association with LDL, a molecular feature that may also be responsible for the association between PCSK9 and HDL.^22^ The lipid remodeling occurring upon HDL during the postprandial phase could therefore alter the binding affinity of PCSK9. We, alongside other reports using NMR-based lipoprotein analyses, reveal a reduction in S.HDL particle numbers over the postprandial response.^23^ Interestingly, S.HDL was the subpopulation of HDL found to be enriched in PCSK9. A positive correlation of PCSK9 with the particle number and lipid content of S.HDL was of similar strength as that with apoB-carrying lipoproteins. The release of both PCSK9 and apoC3 from HDL during postprandial lipemia may result in the synergistic regulation not only of hepatic LDLR abundance but also the catabolic turnover of TRLs. PCSK9 has been shown to regulate the production of TRLs in both the liver and intestine, through an LDLR-dependent and independent manner.^24–26^ It could be envisaged that PCSK9 released from HDL, a facilitator of PCSK9-mediated LDLR degradation, may bind apoB-containing lipoproteins that are known to inhibit PCSK9 function and, therefore, control the hepatic uptake of triglycerides in the postprandial phase. Furthermore, females have a greater postprandial shift in HDL subpopulation redistribution when compared with men, a feature that may drive alterations in the postprandial clearance of lipoproteins due to female HDL being enriched in PCSK9.^23^

### HDL and PCSK9 Function

The core function of PCSK9, and the rationale for therapeutic targeting, is its downregulation of hepatic LDLR surface expression, thereby raising circulating levels of atherogenic apoB-containing lipoprotein particles.^27^ Heparan sulfate proteoglycans capture circulating PCSK9, thus presenting PCSK9 to the LDLR on the hepatocyte surface.^28^ HDL is a known binder of heparan sulfate proteoglycans.^28,29^ The increase in cellular uptake of PCSK9 in the presence of HDL potentially reveals a previously unidentified, LDLR-independent uptake mechanism of PCSK9 that could be reliant upon cell surface proteoglycans. Treatment of HepG2 cells with either PCSK9 or HDL led to an internalization of SDC1 and SDC2 from the cell surface. PCSK9 not only regulates the LDLR through binding to the extracellular region of this receptor but can also control LDLR degradation within the cell.^30^ Unlike LDL that is thought to inhibit PCSK9 function upon the LDLR,^3^ our data suggest, that at physiological concentrations of PCSK9 and HDL, HDL promotes the multimerization of PCSK9 in a dose-dependent manner. The multimeric state of PCSK9 has previously been associated with its LDLR degrading capabilities, therefore a varying ratio of LDL and HDL within the human circulation could determine the activity of PCSK9, which is further supported by the distinct promotion of multimerization of PCSK9 by HDL and LDL in our study.^31^ An interesting observation made by Fan et al^31^ is that the regulation of PCSK9 multimerization by lipoproteins may be dependent upon the apoE content of a given lipoprotein, pertinent to our study in which PCSK9 and apoE form a functional cluster (Figure 5).

### Clinical Implications

The measurement of plasma PCSK9 is a poor marker of activity, whereby circulating PCSK9 levels account for only 7% of the variation in plasma LDL-C (LDL cholesterol), hence, total PCSK9 levels do not provide added benefit when used as a biomarker for CVD risk.^16,32^ The evident observations that lipoproteins alter PCSK9 function prompt future work to determine whether lipoprotein characteristics alter the therapeutic efficacy of PCSK9 inhibitors, that is, whether the role of sex on HDL-PCSK9 might impact the efficacy of PCSK9 inhibition. Before our recent observation that PCSK9 binds primarily to HDL in the human circulation, it was thought that PCSK9 is bound to LDL (20%–50%) and that LDL can sequester and inhibit PCSK9 action upon the LDLR in vitro.^3,5^ The interaction between HDL and PCSK9 creates the possibility that a region targetable by a small molecule may be identified that could be administered orally to inhibit PCSK9 activity instead of monoclonal antibodies, which require regular injections and are expensive.

### Limitations

The study of lipoproteins and their protein constituents relies upon their purity, which is dependent upon the isolation method used. Ultracentrifugation methods, although favored, result in the destabilization and loss of labile interactions, as we observe in the case of PCSK9. Second, Lp(a) is known to overlap in densities with HDL, therefore, resulting in the presence of apoB and apo(a) within HDL samples isolated by density techniques. Although apoB presence could be deemed a contaminant within HDL, our XLMS approach identified an interaction between apoA1 and apoB, prompting the need to further interrogate the structural basis for the interaction between lipoproteins, in the facilitation of lipid and protein transfer.

### Conclusions

The present study provides evidence that PCSK9 is a core member of the HDL proteome and that this association is altered during the postprandial response. HDL increases PCSK9 activity and may link PCSK9 activity to circulating lipids levels. Further elucidation of the interactions between circulating lipoproteins and PCSK9 might reveal new avenues of therapeutic targeting of PCSK9.

## Article Information

### Acknowledgments

We would like to thank all individuals who participated in the studies presented. We would also like to thank Ella Reed and Dr Abhishek Joshi for critical proofreading of the manuscript. Finally, we would like to thank the Nikon Imaging Centre at King’s College London.

### Sources of Funding

M. Mayr is a British Heart Foundation (BHF) Chair Holder (CH/16/3/32406) with BHF programme grant support (RG/16/14/32397). The research was also supported by the National Institute for Health Research (NIHR) Biomedical Research Centre based at Guy’s and St Thomas’ National Health Service (NHS) Foundation Trust and King’s College London (the views expressed are those of the author(s) and not necessarily those of the NHS, the NIHR, or the Department of Health), and the Deutsche Forschungsgemeinschaft, Sonderforschungsbereich 1116, Project B11 to B. Levkau, Institute for Molecular Medicine III, University Hospital Düsseldorf, Heinrich Heine University, Düsseldorf, Germany. The Bruneck Study is supported by the Pustertaler Verein zur Vorbeugung von Herz- und Hirngefässerkrankungen, the Gesundheitsbezirk Bruneck, and the Sanitätsbetrieb Südtirol, province of Bolzano, Italy, and received support from the excellence initiative (Competence Centers for Excellent Technologies—COMET) of the Austrian Research Promotion Agency (FFG, K-Project No. 843536) funded by the Federal Ministry for Transport Innovation and Technology (BMVIT), Federal Ministry of Education, Science and Research (BMBWF), Wirtschaftsagentur Wien, Wirtschafts- und Forschungsförderung Salzburg and Standortagentur Tirol. M. Mayr is also supported by the Leducq Foundation (18CVD02) and the Research Center on Vascular Ageing and Stroke (VASCage, No. 868624). As a COMET center, VASCage is funded within the COMET program—Competence Centers for Excellent Technologies by the Austrian Ministry for Climate Action, Environment, Energy, Mobility, Innovation, and Technology, the Austrian Ministry for Digital and Economic Affairs, and the federal states Tyrol, Salzburg, and Vienna. The postprandial work (W.L. Hall., S.E. Berry) was supported by a grant from Biotechnology and Biological Sciences Research Council Diet and Health Research Industry Club (BBSRC DRINC) (BB/N020987/1).

### Disclosures

S.A. Burnap and M. Mayr are named inventors on a patent application filed by King’s College London for PCSK9 (proprotein convertase subtilisin/kexin type 9) measurements. S. Tsimikas is a coinventor and receives royalties from patents owned by UCSD on oxidation-specific antibodies and of biomarkers related to oxidized lipoproteins and is a co-founder and has an equity interest in Oxitope, Inc, and its affiliates (Oxitope) as well as in Kleanthi Diagnostics, LLC (Kleanthi). Although these relationships have been identified for conflict of interest management based on the overall scope of the project and its potential benefit to Oxitope and Kleanthi, the research findings included in this particular publication may not necessarily relate to the interests of Oxitope and Kleanthi. The terms of this arrangement have been reviewed and approved by the University of California, San Diego in accordance with its conflict of interest policies. S.E. Berry is a consultant to ZOE Ltd. The other authors report no conflicts.

### Supplemental Materials

Expanded Materials and Methods

Data Supplement Figures I–XIV

Data Supplement Tables I–VI

References 33–41

## Supplementary Material



## Novelty and Significance

### What Is Known?

- PCSK9 (proprotein convertase subtilisin/kexin type 9) promotes the lysosomal degradation of the LDLR (low-density lipoprotein receptor).
- PCSK9 circulates in a free and lipoprotein-bound form.
- LDL can act to inhibit PCSK9 yet the functional consequence of the interaction between PCSK9 and HDL (high-density lipoprotein) is unknown.
