# Supplementary figures and images for: PCSK9 Activity Is Potentiated Through HDL Binding
Source: Circ Res. 2021 Oct 4;129(11):1039–53. doi: 10.1161/CIRCRESAHA.121.319272 (PMC8579991; doi:10.1161/CIRCRESAHA.121.319272)

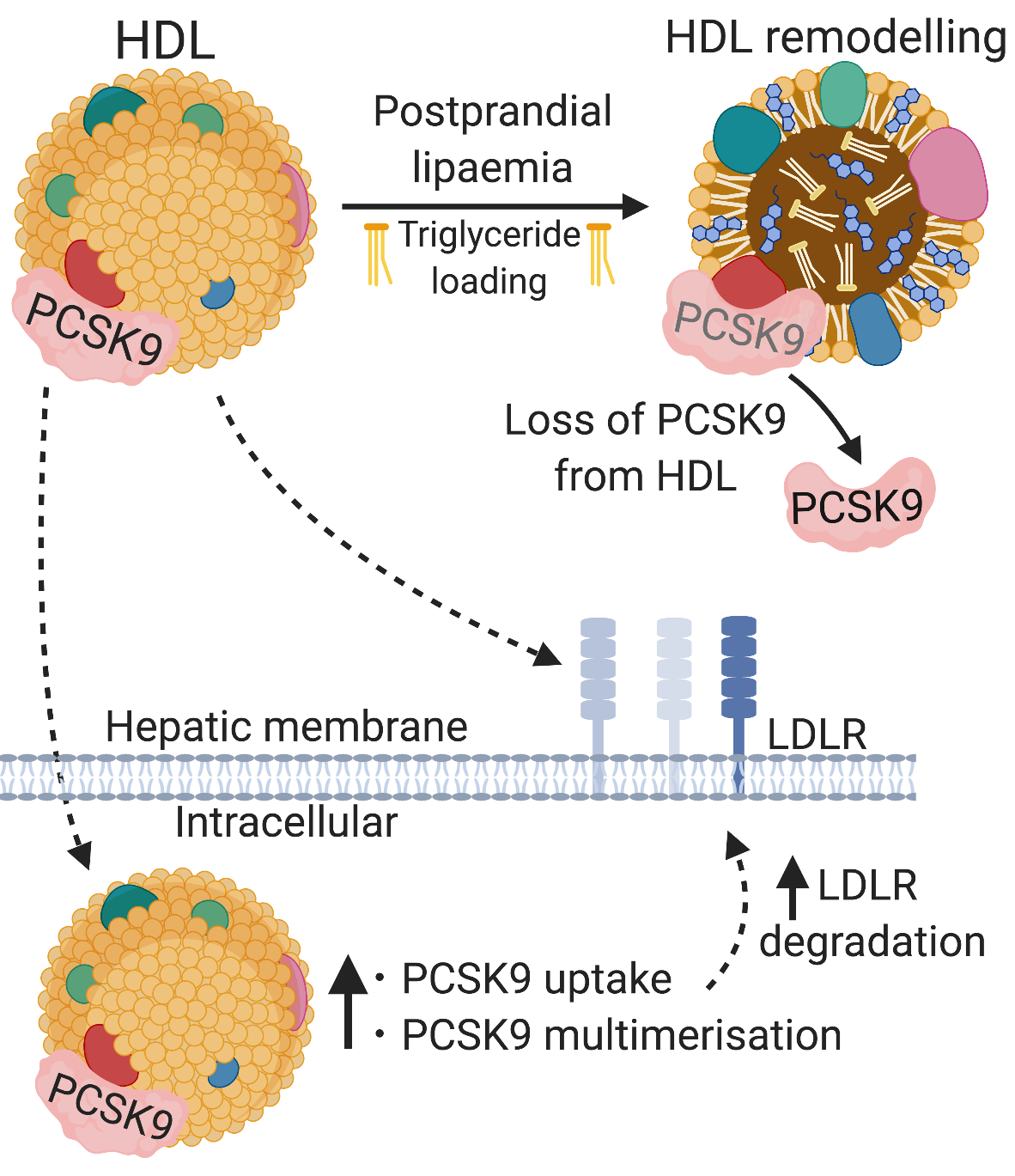

Supplement: Supplementary file 1 [file res-129-1039-s001.png]
