# Supplementary material for: PCSK9 Activity Is Potentiated Through HDL Binding
Source: Circ Res. 2021 Oct 4;129(11):1039–53. doi: 10.1161/CIRCRESAHA.121.319272 (PMC8579991; doi:10.1161/CIRCRESAHA.121.319272)

Figure 4B

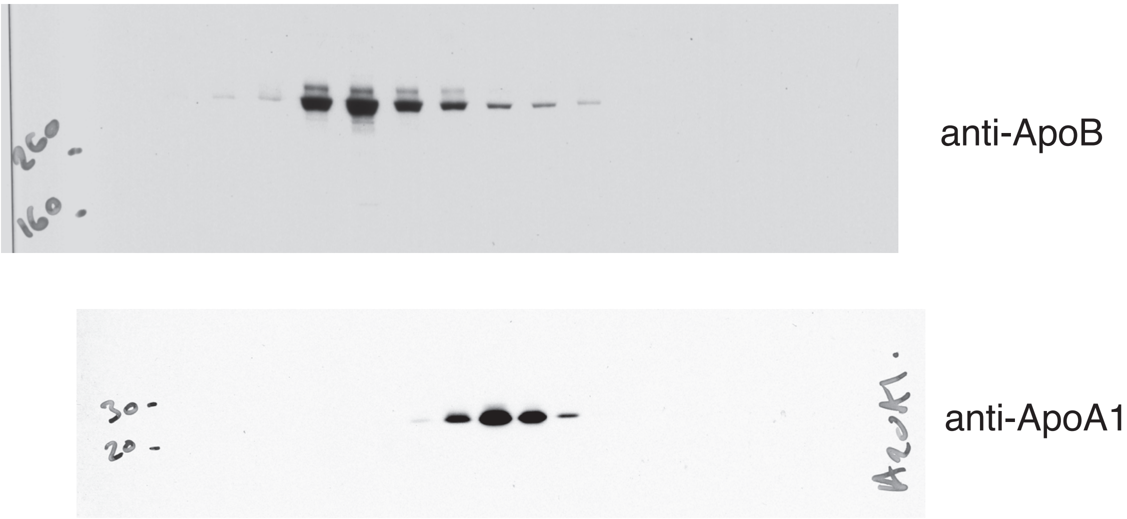

Figure 4C

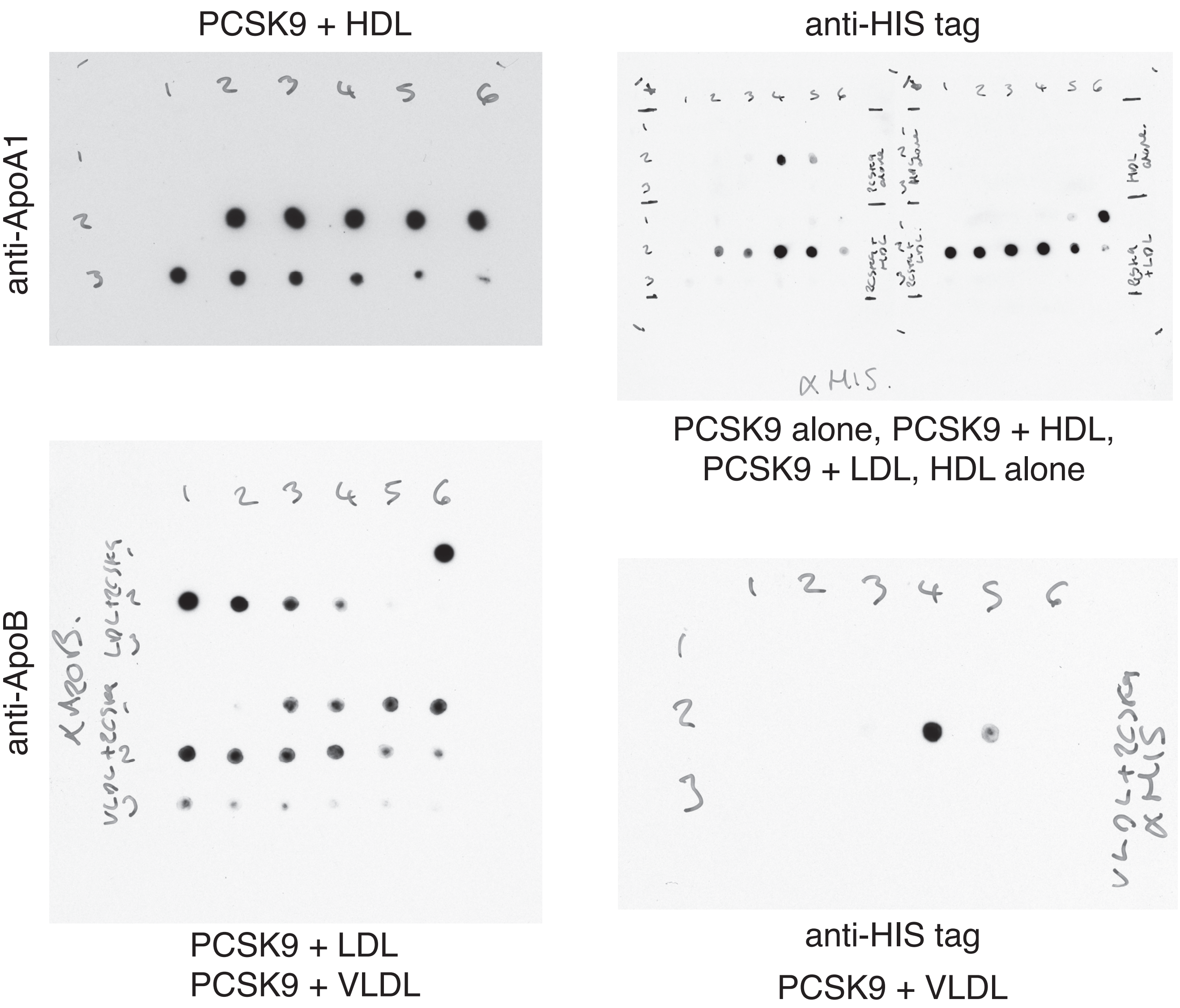

Figure 4D

anti-ApoA1: Bound fraction

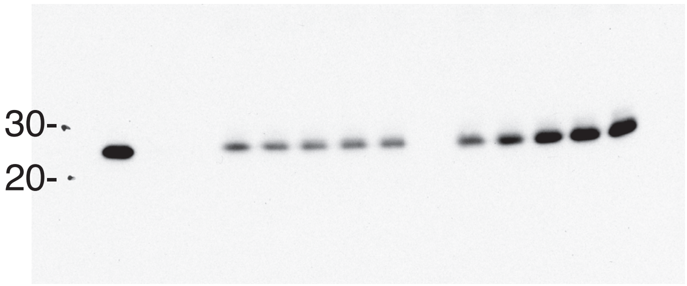

anti-ApoA1: S/N

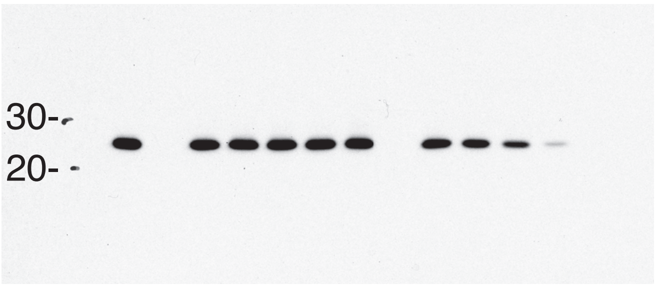

Figure 4F

anti-ApoA1

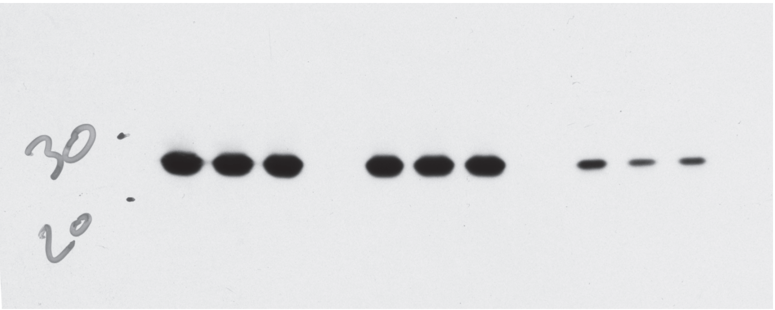

Figure 6B

anti-Beta Actin

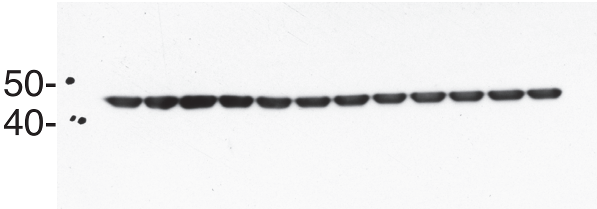

anti-ApoA1

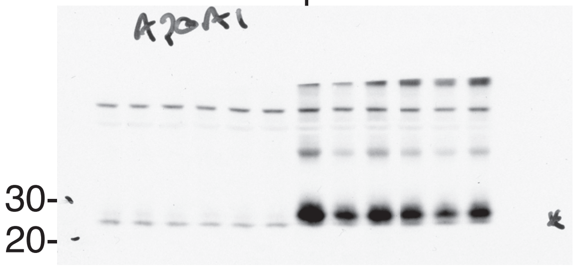

anti-HIS tag
PCSK9 + rHDL

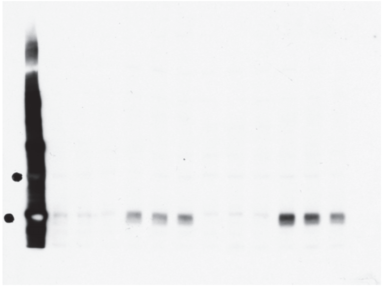

anti-HIS tag
PCSK9 + ucHDL

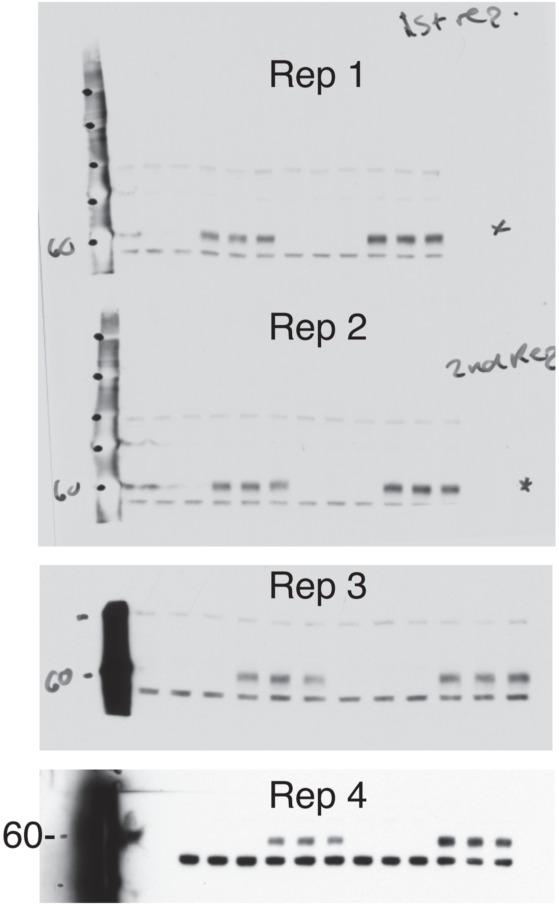

Figure 6D

anti-HIS tag

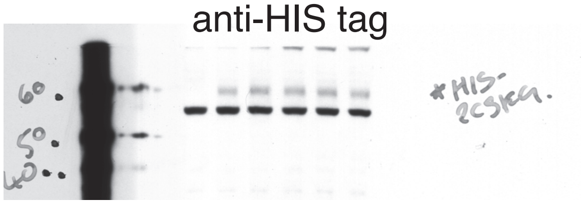

anti-ApoB

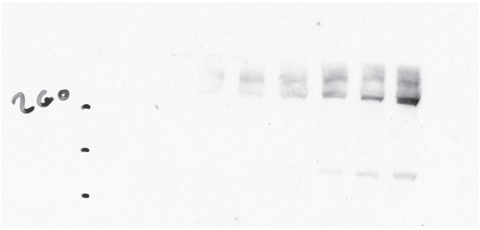

Figure 6E

anti-HIS tag

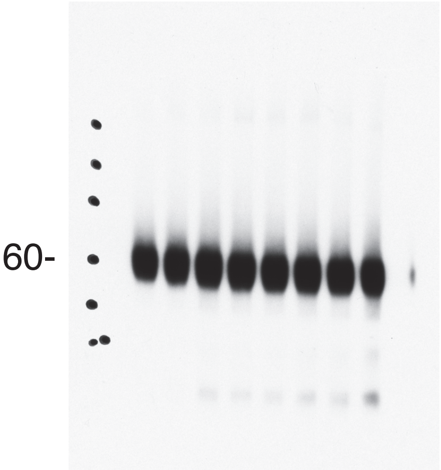

rHDL + PCSK9

anti-HIS tag

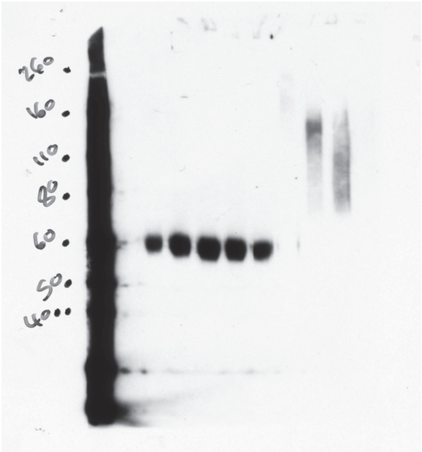

ucHDL + PCSK9

Figure 7A

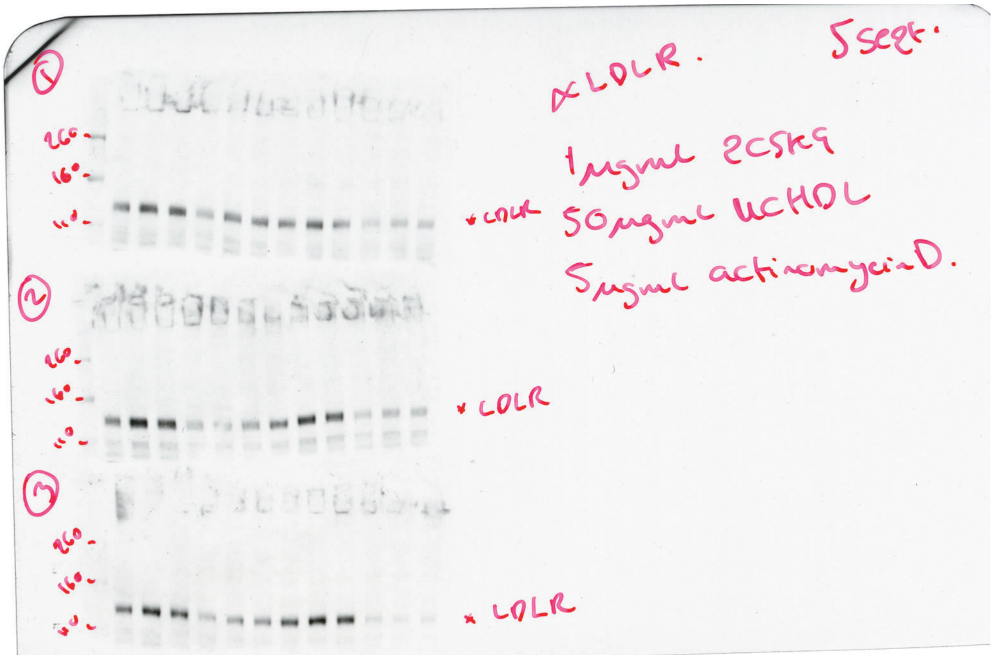

anti-LDLR
3 reps

anti-PCSK9 multimer quantification

Rep 1

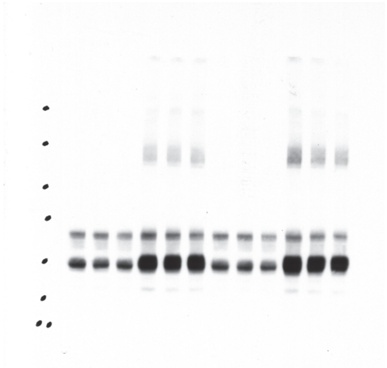

Rep 2

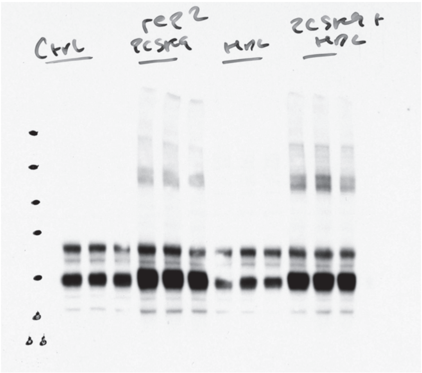

Rep 3

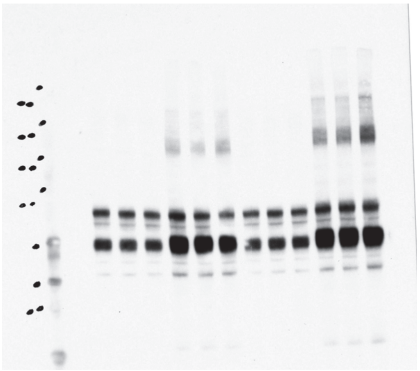

anti-HIS tag

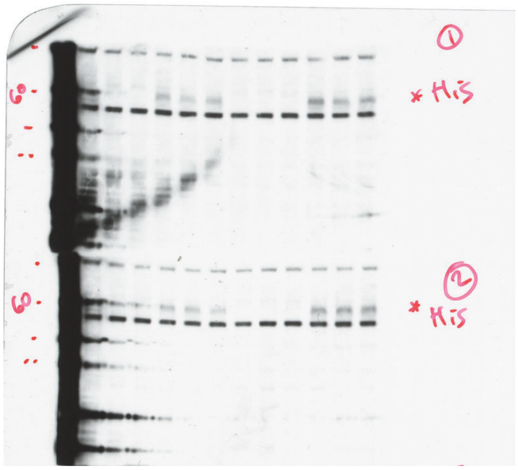

Rep 1

Rep 2

60-

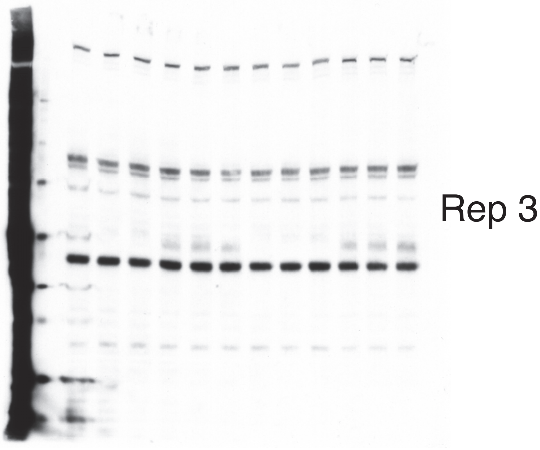

Rep 3

Figure 7A continued

anti-ApoA1

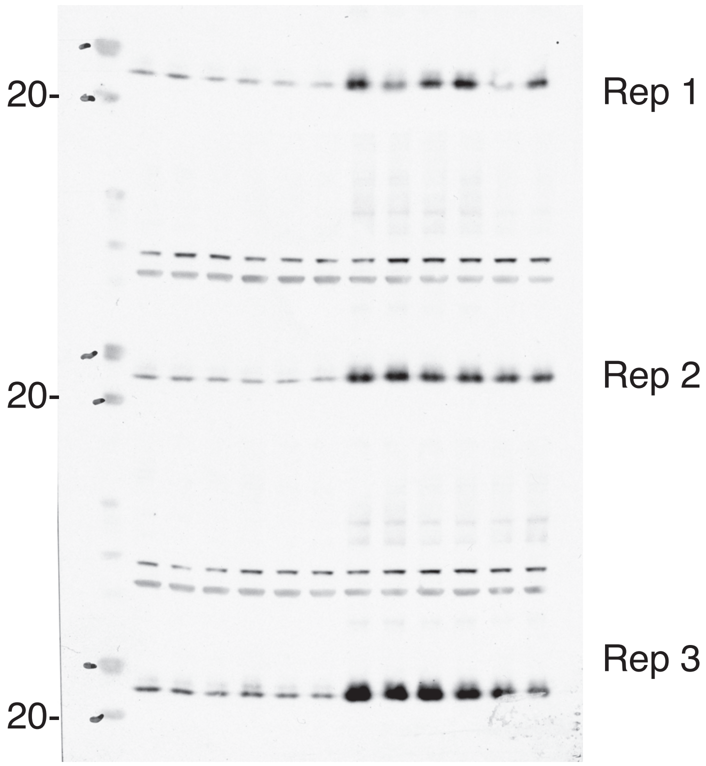

anti-Beta actin

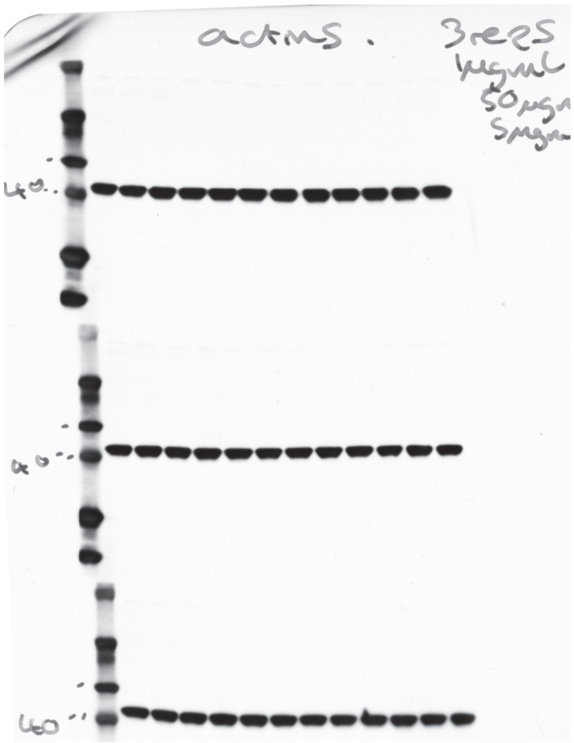

Figure 7C

anti-LDLR

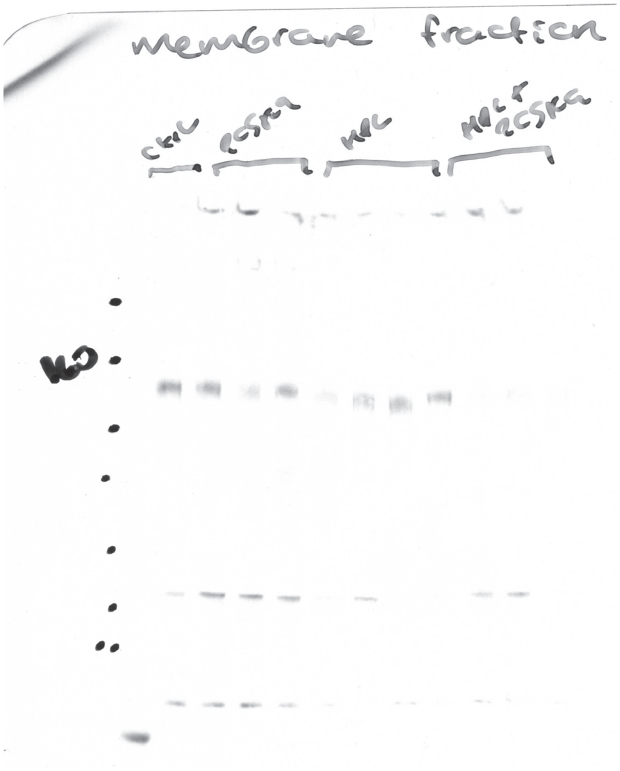

anti-TfR

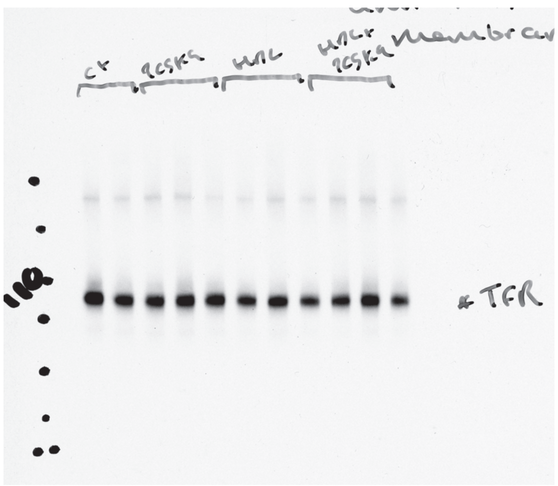

Online Figure V

anti-ApoA1: PCSK9 + ucHDL

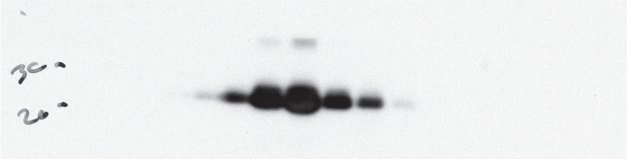

anti-PCSK9: PCSK9 alone

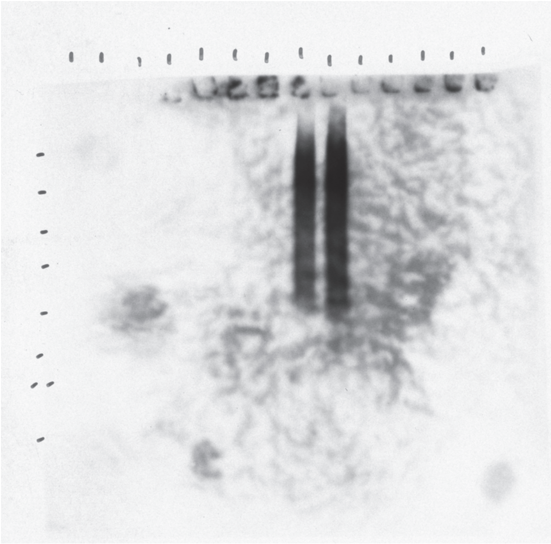

anti-PCSK9: PCSK9 + ucHDL

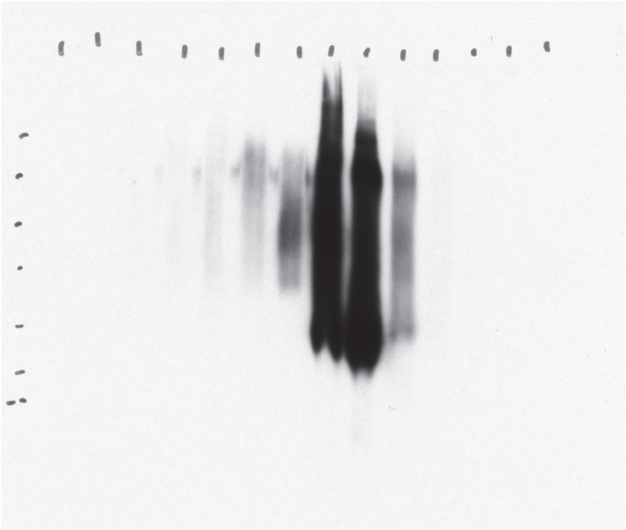

Online Figure XII

anti-PCSK9: PCSK9 + increasing ucHDL

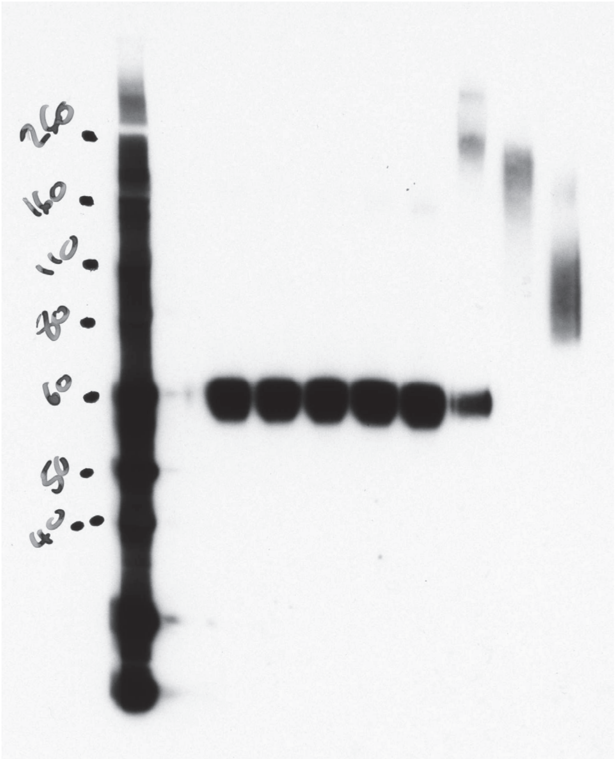

anti-PCSK9:
PCSK9 + increasing ucLDL

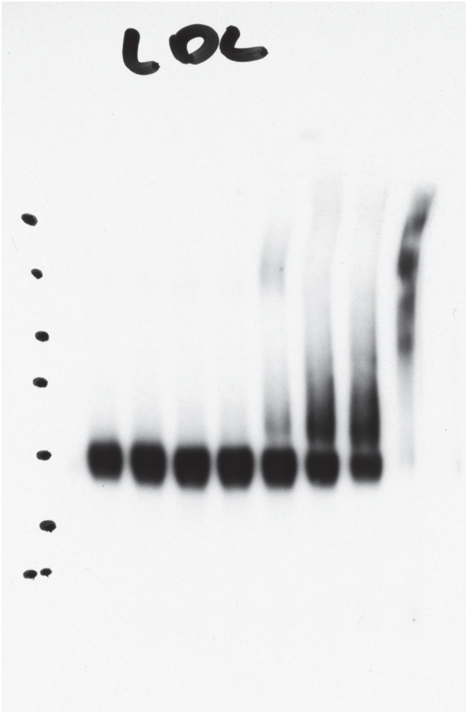

Online Figure XIII

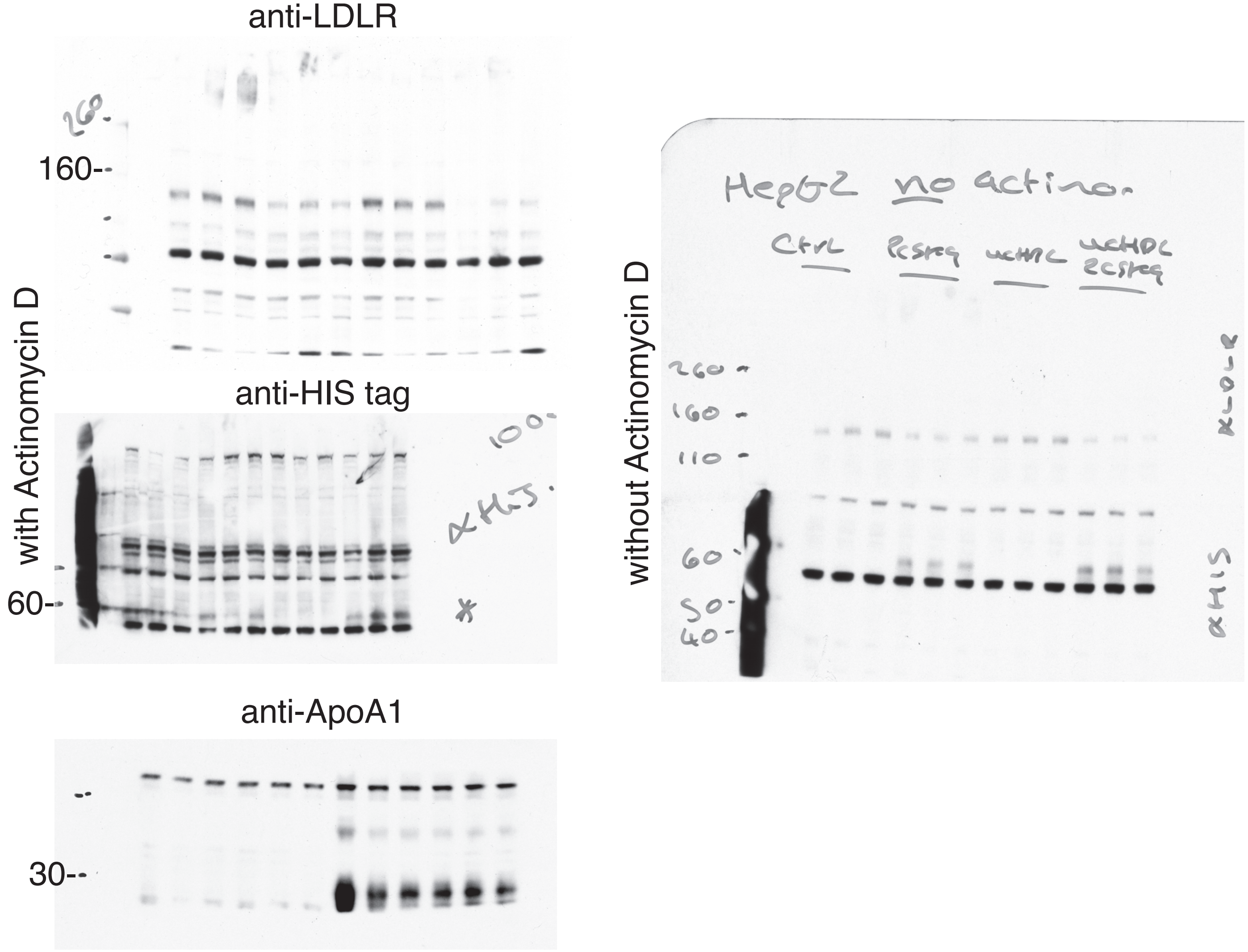

Online Figure XIV- without Actinomycin D

anti-LDLR: PCSK9 dose

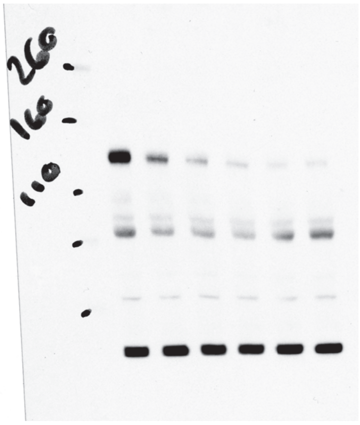

anti-PCSK9: PCSK9 dose

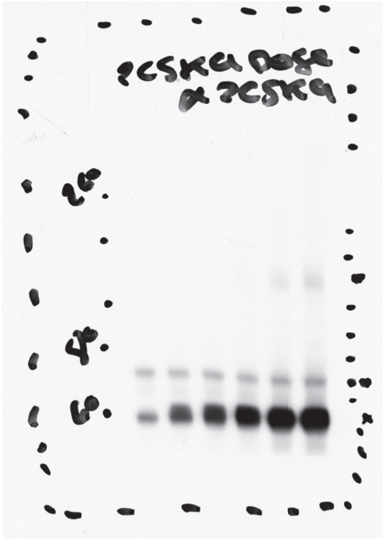

anti-LDLR: rHDL dose

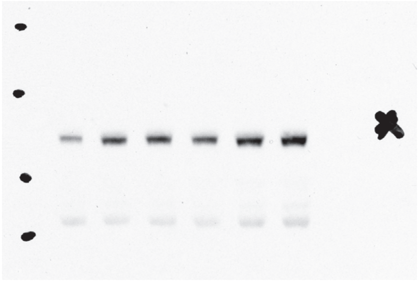

anti-ApoA1: rHDL dose

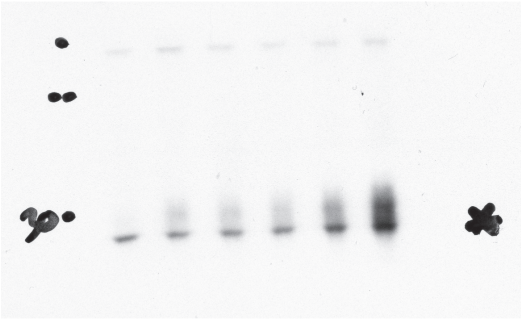

anti-LDLR: ucHDL dose

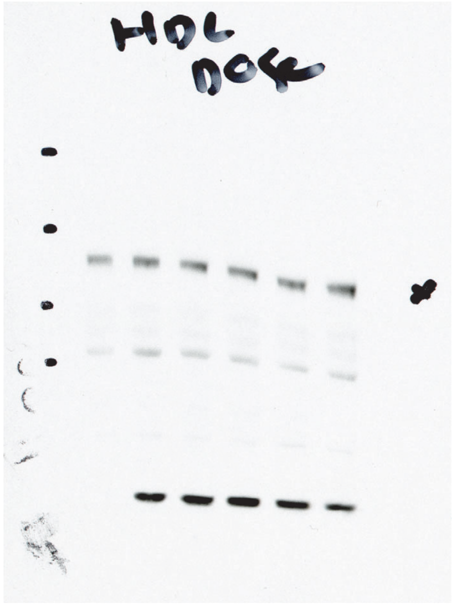

anti-ApoA1: ucHDL dose

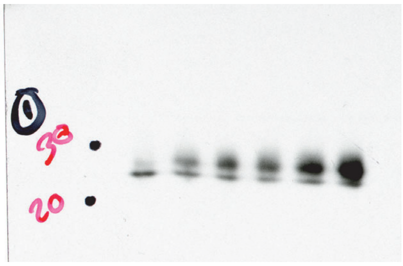

anti-Beta actin:
ucHDL dose

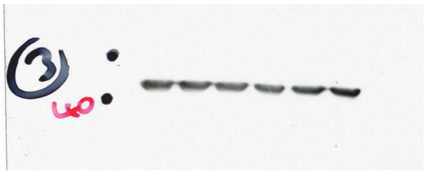

Online Figure XIV- with Actinomycin D

anti-HIS tag: PCSK9 dose

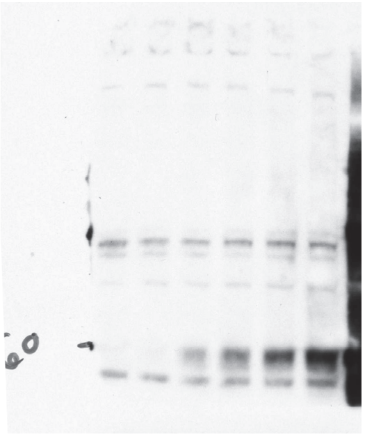

anti-LDLR: PCSK9 dose

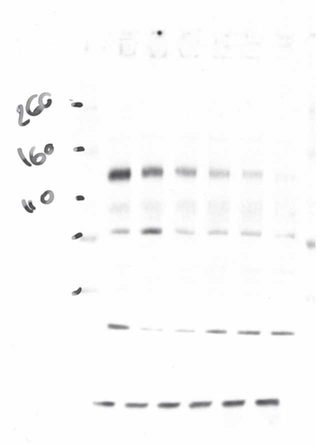

anti-Beta actin: PCSK9 dose

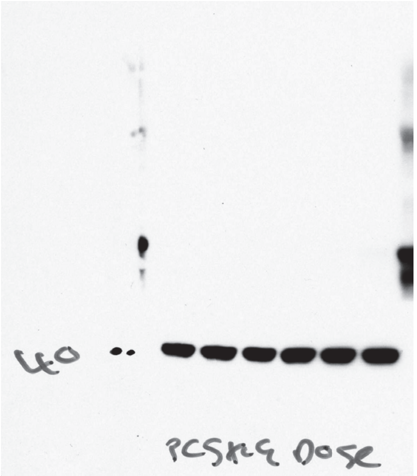

anti-ApoA1: rHDL dose

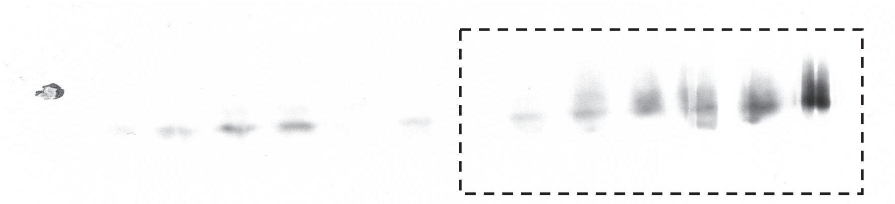

anti-LDLR: rHDL dose

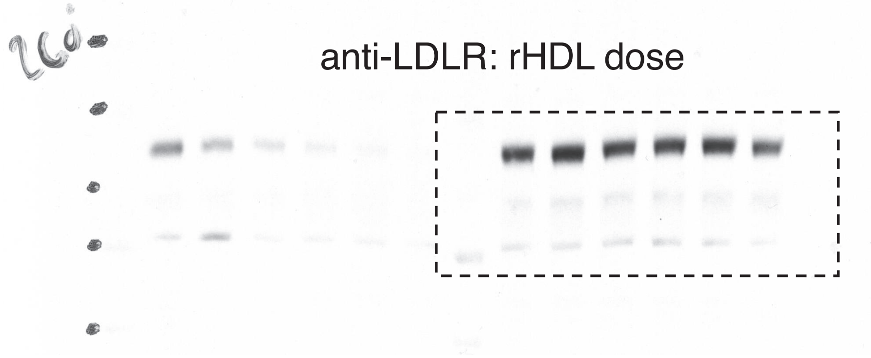

anti-Beta actin: rHDL dose

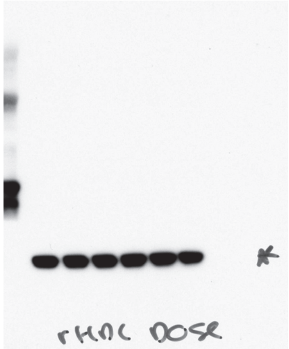

anti-LDLR: ucHDL dose

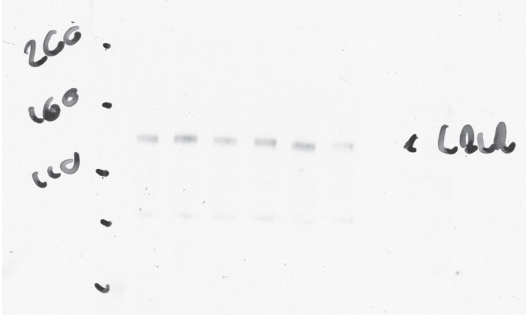

anti-ApoA1: ucHDL dose

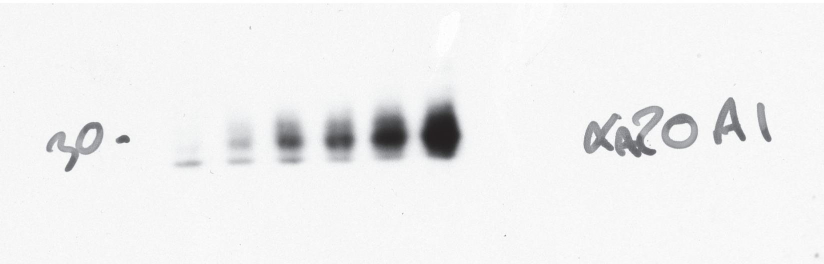

anti-Beta actin: ucHDL dose

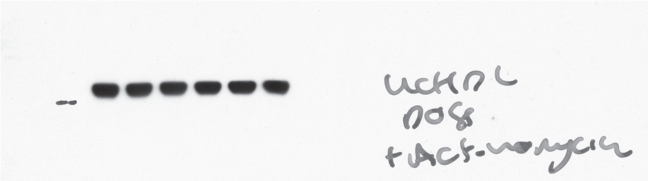

Supplement: Supplementary file 4 [file res-129-1039-s004.pdf]
