# Supplementary material for: PCSK9 Activity Is Potentiated Through HDL Binding
Source: Circ Res. 2021 Oct 4;129(11):1039–53. doi: 10.1161/CIRCRESAHA.121.319272 (PMC8579991; doi:10.1161/CIRCRESAHA.121.319272)
Supplement: Supplementary file 5 [file res-129-1039-s005.pdf]

Major Resources Table

In order to allow validation and replication of experiments, all essential research materials listed in the Methods should be included in the Major Resources Table below. Authors are encouraged to use public repositories for protocols, data, code, and other materials and provide persistent identifiers and/or links to repositories when available. Authors may add or delete rows as needed.

Animals (in vivo studies)

Species	Vendor or Source	Background Strain	Sex	Persistent ID / URL

Genetically Modified Animals

	Species	Vendor or Source	Background Strain	Other Information	Persistent ID / URL
**Parent - Male**					
**Parent - Female**					

Antibodies

Target antigen	Vendor or Source	Catalog #	Working concentration	Lot # (preferred but not required)	Persistent ID / URL
PCSK9	Abcam	ab181142	1:1000		https://www.abcam.com/pcsk9-antibody-epr76272-ab181142.html
ApoA1-sepharose conjugation	Acedemy Bio-Medical	11A-G2b			https://www.academybiomed.com/collections/antibody/products/a03-goat-anti-human-apolipoprotein-ai-polyclonal-antibody?variant=22226734186575
ApoA1	Abcam	Ab52945	1:1000		https://www.abcam.com/apolipoprotein-a-i-antibody-ep1368y-ab52945.html
ApoB	Mouse	MIA1609	1:1000		https://www.thermofisher.com/antibody/MIA1609
LDLR	Rabbit	ab52818	1:1000		https://www.abcam.com/ldl-receptor-antibody-ep1553y-ab52818.html
Transferrin	Mouse	ab269513	1:1000		https://www.abcam.com/transferrin-receptor-antibody-h684-ab269513.html
6x His tag	Mouse	ab18184	1:1000		https://www.abcam.com/6x-his-tag-antibody-hish8-ab18184.html
β-Actin	Mouse	A1978	1:10000		https://www.sigmaaldrich.com/GB/en/product/sigma/a1978
HRP-anti-Rabbit	Mouse	211-032-171	1:5000		https://www.jacksonimmuno.com/catalog/products/211-032-171
HRP-anti-Mouse	Goat	115-035-174	1:5000		https://www.jacksonimmuno.com/catalog/products/115-035-174

DNA/cDNA Clones

Clone Name	Sequence	Source / Repository	Persistent ID / URL

Cultured Cells

Name	Vendor or	Sex (F, M, or	Persistent ID / URL

DOI [to be added]

	Source	unknown)	
HepG2	European Collection of Authenticated Cell Cultures (ECACC)	Male	https://www.phe-culturecollections.org.uk/products/celllines/generalcell/detail.jsp?refId=85011430&collection=ecacc_gc

Data & Code Availability

Description	Source / Repository	Persistent ID / URL

Other

Description	Source / Repository	Persistent ID / URL
Human PCSK9 ELISA Duoset Kit	R&D	https://www.rndsystems.com/products/human-proprotein-convertase-9-pcsk9-duoset-elisa_dy3888
AbsoluteIDQ® p400 HR kit	Biocrates	https://biocrates.com/absoluteidq-p400-hr-kit/
High Density Lipoprotein (HDL) Depletion Column	Genway Biotech	https://www.genwaybio.com/affinity-purified-anti-human-high-density-lipoprotein-hdl-igy-gel-kit
SuperoseTM 6 Increase 10/300 GL column	Cytiva	https://www.cytivalifesciences.com/en/us/support/products/superose-6-10-300-gl-17517201#:~:text=Superose%206%2010%2F300%20GL,5000%20to%205%20000%20000.
TSKgel G5000/G4000PWXL	Tosoh Bioscience	https://www.separations.eu.tosohbioscience.com/solutions/hplc-products/size-exclusion/aqueous-size-exclusion-gfc/tskgel-pwxl-columns/tskgel-g4000pwxl

DOI [to be added]
