# Supplementary material for: PCSK9 Activity Is Potentiated Through HDL Binding
Source: Circ Res. 2021 Oct 4;129(11):1039–53. doi: 10.1161/CIRCRESAHA.121.319272 (PMC8579991; doi:10.1161/CIRCRESAHA.121.319272)
Supplement: Supplementary file 6 [file res-129-1039-s006.pdf]

Online Supplemental Material

PCSK9 activity is potentiated through HDL binding

Sean A. Burnap[1], Katherine Sattler[2], Raimund Pechlaner[3], Elisa Duregotti[1], Ruifang Lu[1], Konstantinos Theofilatos[1], Kaloyan Takov[1], Gerd Heusch[2], Sotirios Tsimikas[4], Carlos Fernández-Hernando[5], Sarah E. Berry[6], Wendy Hall[6], Marlene Notdurfter[7], Gregorio Rungger[3], Bernhard Paulweber[8], Johann Willeit[3], Stefan Kiechl[3,9], Bodo Levkau[10†], Manuel Mayr[1†*].

[1] King's College London British Heart Foundation Centre, School of Cardiovascular Medicine and Sciences, London, United Kingdom

[2] Institute for Pathophysiology, University Hospital Essen, West German Heart and Vascular Center, Essen, Germany

[3] Department of Neurology, Medical University of Innsbruck, Austria

[4] Division of Cardiovascular Medicine, University of California San Diego, La Jolla, CA, USA

[5] Vascular Biology and Therapeutics Program, Yale University School of Medicine, New Haven, USA

[6] Department of Nutritional Sciences, School of Life Course Sciences, Faculty of Life Sciences & Medicine, King's College London, United Kingdom

[7] Department of Internal Medicine, Hospital of Bruneck, Italy

[8]First Department of Medicine, Paracelsus Medical University Salzburg, 5020 Salzburg, Austria

[9] VASCage - Research Centre on Vascular Ageing and Stroke, Innsbruck, Austria

[10] Institute for Molecular Medicine III, Heinrich-Heine-University, Medical Faculty, Düsseldorf, Germany

† Joint senior authorship.

*To whom correspondence should be addressed: Manuel Mayr; King's British Heart Foundation Centre, King's College London, 125 Coldharbour Lane, London, SE59NU UK; Phone: +44(0)2078485446; +44(0)2078485298; Email: manuel.mayr@kcl.ac.uk

Online Supplemental Methods containing references 33-41

Online Supplemental Tables:

Online Table I. Plasma PCSK9 NMR correlations in Bruneck.

Online Table II. Postprandial alterations over time.

Online Table III. HDL proteome alterations over the postprandial time course.

Online Table IV. Sex-driven HDL proteome alterations: TMT quantification.

Online Table V. Sex-driven HDL proteome alterations: Label-free quantification.

Online Table VI. HepG2 cell surface protein changes upon PCSK9 and ucHDL treatment.

Online Supplemental Figures:

Online Figure I. PCSK9 is enriched in small-HDL.

Online Figure II. Postprandial HDL proteome remodelling.

Online Figure III. XLMS methodological development using BSA.

Online Figure IV. XLMS plasma interactome.

Online Figure V. XLMS plasma interactome.

Online Figure VI. MS-based validation of PCSK9 and lipoproteins used for experiments.

Online Figure VII. PCSK9 plasma levels in response to statin treatment.

Online Figure VIII. Proteomic analysis of HDL: Comparison against published reports.

Online Figure IX. HDL proteome correlations.

Online Figure X. PCSK9 is a member of the HDL proteome in patients with CAD.

Online Figure XI. Smoking drives HDL proteome alteration.

Online Figure XII. HDL and LDL differentially induce PCSK9 multimerisation.

Online Figure XIII. HDL potentiates PCSK9-mediated LDLR degradation.

Online Figure XIV. Actinomycin D ablates HDL-mediated LDLR synthesis.

ONLINE METHODS

Community-based Studies. The Bruneck Study is a prospective, community-based survey of the epidemiology and pathogenesis of atherosclerosis and CVD[33]. An age- and sex-stratified random sample of all inhabitants of Bruneck, Italy, all of Caucasian descent, was enrolled in 1990. In 2000, 702 subjects participated in the second quinquennial follow-up. The study protocol was approved by the ethics committees of Bolzano and Verona, and conformed to the Declaration of Helsinki, and all study subjects gave written informed consent. NMR-based lipoprotein profiling, PCSK9 measurements (DY3888, R&D Systems) and targeted apolipoprotein measurements by MS were conducted on citrate plasma samples (n = 656). The Salzburg Atherosclerosis Prevention Program in Subjects at High Individual Risk (SAPHIR) study is a prospective cohort study conducted in 1,770 healthy unrelated subjects (663 females and 1,107 males aged 39–67 years) who were recruited by health screening programs in large companies in and around the city of Salzburg, Austria[34]. For the current analysis, we used a nested case-control design. We selected all participants with incident primary CVD event (defined as MI, ischemic stroke, or vascular death), all participants with CVD other than stroke and MI, plus an age- and sex-matched subcohort (n=270). Citrate plasma samples were prepared after an overnight fast and 12 hours of abstinence from smoking and aliquots were stored at –80°C.

Nuclear Magnetic Resonance Spectroscopy (NMR). NMR-based lipoprotein profiling was conducted using the commercial Nightingale Health assay (Nightingale Health Ltd). This metabolic profiling platform enables the quantification of 14 lipoprotein subclasses defined as follows: extremely large-VLDL (>75 nm), five subclasses of VLDL (average particle diameter of 64.0 nm, 53.6 nm, 44.5 nm, 36.8 nm and 31.3 nm), intermediate density lipoprotein (IDL) (28.6 nm), three LDL subclasses (25.5 nm, 23.0 nm and 18.7 nm) and lastly four HDL subclasses (14.3 nm, 12.1 nm, 10.9 nm and 8.7 nm). The particle number of each lipoprotein subclass is quantified alongside lipid content. This NMR-based platform has been used previously in multiple epidemiological studies, where detailed technological information can be found[35].

Multiple Reaction Monitoring-based Proteomics. Targeted quantitation of plasma proteins was conducted using the commercially available Plasma Dive kit (Biognosys). Plasma samples were processed according to the manufacturer's instructions[36]. Briefly, 10 μL of plasma was denatured and reduced in 90 μL of denature buffer and alkylated by the addition of 16 μL alkylation solution. 3 μL of alkylated protein (approximately 20 μg) were spiked with authentic heavy peptide standards (the peptide standard for ApoB-100 did not overlap with the proximal portion of ApoB that would include both ApoB-48 and ApoB-100). An in-solution tryptic digestion (Pierce Porcine Tryspin, enzyme: protein = 1:50, Thermo Fisher) was performed overnight at 37°C with shaking. Digestion was stopped by the addition of 10 μL, 10% trifluoroacetic acid (TFA). After solid-phase extraction with C18 cartridges (Bravo AssayMAP, Agilent Technologies), the eluted peptides were dried using a

SpeedVac (Thermo Fisher Scientific) and resuspended in 40 μL of liquid chromatography solution.

Peptides were analysed on an Agilent 1290 Infinity II liquid chromatography system (Agilent Technologies) interfaced to an Agilent 6495 Triple Quadrupole MS (Agilent Technologies). Both instruments were controlled by MassHunter Workstation software (version B.08.00). Samples (10 μL) were directly injected onto a 25-cm column (AdvanceBio Peptide Mapping, C18, 2.1mm×250 mm, 2.7 μm, 120 Å, Agilent Technologies) and separated over a 23-minute gradient at 350 μL/min. Data files were analysed using SpectroDive 8 (Biognosys). Every peak integration was manually checked. Q-value <0.01 (FDR <1%) was used. The absolute concentration was calculated using Light/Heavy peptide signal intensity and known heavy peptide concentration.

Enzyme-Linked Immunosorbent Assay (ELISA). Human PCSK9 concentrations were measured using the DuoSet ELISA Development kit (DY3888, R&D Systems) and the corresponding DuoSet Ancillary Reagent Kit 2 (R&D Systems) according to the manufacturer's instructions. Absorbance at 450 nm was measured on a plate reader (Tecan Infinite 200 Pro) using 570 nm as a reference wavelength. Concentrations were calculated using a 4-parameter logistic (4-PL) fit.

Postprandial Studies. Postprandial samples were analysed from a double-blinded, 3-armed, randomised controlled trial (trial registration; clinicaltrials.gov NCT03191513; approved by King's College London Research Ethics Committee (HR-16/17-4397)) in healthy adults (n=20; 10 men, 10 women) aged 58 (SD 6.4) years. Samples were selected following consumption of the control test meal only containing 50 g rapeseed oil (61% 18:1n-9*cis*; 19% 18:2n-6*cis*) fed in the form of a muffin and a

milkshake (to deliver 897 kcal, 50 g fat, 18 g protein, 88 g carbohydrate), following an overnight fast, a 50 g fat load has been shown to be the optimum quantity to discriminate between individual postprandial responses. Venous blood samples were collected at hourly intervals 0-8 h postprandially for analysis of plasma. Triacylglycerol (TAG) concentrations were measured on a Siemens ADVIA 1800 using the ADVIA chemistry TG method based on the Fossati three-step enzymatic reaction with a Trinder endpoint. A second, postprandial validation cohort was assessed (n=20, 8 time points), adhering to the same study design and test meal outlined above. Ethical approval for the study (ISRCTN20774126) was obtained from the relevant research ethics committees in the United Kingdom (NREC 08/H1101/122) and the Netherlands (MEC 09-3-009), and written informed consent was given by participants.

HDL-immunoisolation. HDL was immuno-isolated from plasma using human HDL-specific IgY affinity columns according to manufacturer's instructions (Genway Biotech). Briefly, 40 μL of plasma was diluted 10-fold in 360 μL TBS buffer (10 mM Tris, 150 mM NaCl, pH 7.4). Diluted plasma was then added to TBS equilibrated antibody beads and incubated at room temperature with end over end rotation for 15 minutes. Flow through, HDL-depleted plasma, was then collected through centrifugation at 500 x g. The removal of non-specifically bound proteins from the antibody beads was achieved using 500 μL of wash buffer (TBS, 0.05% Tween-20) a total of 3 times. HDL was then stripped from the antibody beads by the addition of 500ul stripping buffer (0.1 M Glycine, pH 2.5), twice. The antibody columns were then regenerated using a series of stripping buffer wash steps, followed by the addition of neutralisation buffer (100 mM Tris-HCl, pH 8.0) and lastly the resuspension in 500 μL TBS containing 0.02% sodium azide for storage. Isolated HDL samples were further

concentrated, due to the large isolation volume, and stored at –80°C until further processing.

Immunoblotting. Laemmli sample buffer (4x) (62.5mM Tris-HCL, pH 6.8, 10% glycerol, 1% SDS, 0.005% bromophenol blue and 10% 2-mercaptoethanol) or without 2-mercaptoethanol was mixed with protein samples and boiled at 95°C for 10 minutes. Protein samples were separated using either 4-12% Bis-Tris or 3-8% Tris-Acetate gradient gels (Thermo scientific) in MOPS and Tris-Acetate SDS running buffer, respectively (Thermo Scientific), at 130V for 90 minutes. Gels were either stained for total protein using SimplyBlue Safe Stain (Thermo Fisher) or proteins were transferred onto nitrocellulose membranes in ice-cold transfer buffer (25 mM Tris-base pH 8.3; 192 mM glycine; 20% methanol) at 350mA for 2 h. Ponceau S red staining was used to determine efficient transfer and equal loading before membranes were blocked in 5% fat-free milk powder in phosphate-buffered saline (PBS) containing 0.1% Tween-20 (PBST)(Sigma). Membranes were incubated in primary antibodies made to appropriate concentrations in 5% bovine serum albumin (BSA) in PBST overnight at 4°C. The membranes were then incubated in the appropriate light-chain specific peroxidase-conjugated secondary antibody in 5% milk/PBST. Membranes were then washed for three times in PBST for 15 minutes. Dot blots were conducted through the blotting of 2 μL of sample directly onto nitrocellulose membrane and allowed to completely dry prior to following the blocking and antibody incubation protocol outlined above. Western blots and dot blots were developed using enhanced chemiluminescence (ECL) (GE Healthcare) on photographic films (GE Healthcare). Densitometry analysis was done using the ImageJ analysis software.

Crosslinking-Mass Spectrometry (XLMS). Protein-protein interactions within plasma and HDL were interrogated using the MS-compatible cross-linker disuccinimidyl sulfoxide (DSSO, Thermo Fisher), following a recently published protocol[37]. Pooled plasma and HDL samples were diluted in 20 mM HEPES buffer (pH 7.5) at a concentration of 1 μg/μL. DSSO was prepared as a 50mM stock solution in DMSO and added to the pooled protein mixture at a final concentration of 1 mM for 1 hour at room temperature. DSSO reactions were quenched through the addition of ammonium bicarbonate (pH 7.2) at a final concentration of 20 mM for 15 minutes at room temperature. Optimisation of cross-linker concentrations are required, in the attempt to prevent over-crosslinking of proteins and the subsequent false identification of protein interactions. A titration of DSSO was conducted upon each sample type and the resulting crosslinked proteins were separated by SDS-PAGE prior to total protein stain; as the cross-linker concentration increases a clear shift in protein bands can be visualised, alongside the appearance of higher molecular weight species. To interrogate PCSK9 interaction partners in HDL, ucHDL (80 μg) was spiked with recombinant PCSK9 (20 μg ACRO Biosystems, PC9-H5223) prior to overnight incubation at 4°C, the ucHDL/PCSK9 mix was then crosslinked as described above.

Crosslinked protein samples were then digested and purified as described below prior to fractionation using Pierce™ strong cation exchange (SCX, Thermo Fisher) spin columns or using a SCX Dionex UltiMate 3000 RSLC system (Luna 5 μm, 100 Å, 50 x 2 mm. Phenomenex). Briefly, dried peptides were resuspended in 20% acetonitrile (ACN) containing 0.4% formic acid (FA). SCX spin columns were first conditioned with 100% methanol (MeOH), followed by 1 M ammonium acetate (AmAc) in 20% ACN containing 0.4%FA and two washes of 20%ACN containing 0.4%FA.

Peptides were then loaded onto conditioned resin and washed twice prior to elution with increasing amounts of AmAc in 20% ACN containing 0.4% FA. All centrifugation steps were 2,000 x g for 5 minutes. Resulting peptide fractions were dried using a speed vac (Thermo Fisher, Savant SPD131DDA) and peptide pellets were resuspended in 5% DMSO / 10% FA / 85% H_2O.

In-solution Protein Digestion. Protein samples were denatured by the addition of a final concentration of 6 M urea and 2 M thiourea and reduced by the addition of a final concentration of 10 mM dithiothreitol (DTT) followed by incubation at 37°C for 1 h, 240 rpm. The samples were then cooled down to room temperature before being alkylated by the addition of a final concentration of 50 mM iodoacetamide followed by incubation in the dark for 30 minutes. Pre-chilled (-20°C) acetone (10x volume) was used to precipitate the samples overnight at -20°C. Samples were centrifuged at 14,000 x g for 40 minutes at 4°C and the supernatant subsequently discarded. Protein pellets were dried using a speed vac, resuspended in 0.1 M TEAB buffer, pH 8.0, containing 0.02% ProteaseMax surfactant (in the case for lipoprotein samples) and MS grade Trypsin/Lys-C (Promega Cooperation) (1:25 enzyme: protein) and digested overnight at 37 °C, 240 rpm. An aliquot of peptide digest was taken for TMT-labelling where necessary, while the digestion was stopped for the remaining peptide for label-free and XLMS analyses by acidification with TFA. Peptides were then purified robotically, using C18 cartridges (Bravo AssayMAP, Agilent Technologies).

Tandem-Mass Tag Peptide Labelling. HDL peptide samples were TMT-11plex labelled according to manufacturer's instructions (Thermo Fisher). Samples and pool were then grouped in equal amounts, ensuring randomisation, and dried

using a speed vac (Thermo Fisher Scientific, Savant SPD131DDA). Grouped TMT samples were then reconstituted with 0.1% TFA in H_2O, for peptide fractionation using high pH reversed-phase spin columns, following manufacturer's instructions (Thermo Fisher).

LC-MS/MS Analysis. The dried peptide samples for label-free and TMT analyses were reconstituted with 0.05% TFA in 2% ACN and separated by a nanoflow LC system (Dionex UltiMate 3000 RSLC nano). Samples were injected onto a nano-trap column (Acclaim® PepMap100 C18 Trap, 5 mm x 300 μm, 5 μm, 100 Å), at a flow rate of 25 μL/min for 3 minutes, using 0.1% FA in H_2O. The following nano-LC gradient was then run at 0.25 μL/min to separate the peptides: 0–10min, 4–10% B; 10–75min, 10–30% B; 75–80min, 30–40% B; 80–85min, 40–99% B; 85–89.8min, 99% B; 89.8–90min, 99–4% B; 90–120min, 4% B; where A = 0.1% FA in H_2O, and B = 80% ACN, 0.1% FA in H_2O. The nano column (EASY-Spray PepMap® RSLC C18, 2µm 100 Å, 75 μm x 50 cm), set at 40°C was connected to an EASY-Spray ion source (Thermo Scientific). Spectra were collected from an Orbitrap mass analyser (Orbitrap Fusion™ Lumos Tribrid, Thermo Scientific) using full MS mode (resolution of 120,000 at 400 m/z) over the mass-to-charge (m/z) range 375–1500. Data-dependent MS2 scan was performed using Quadrupole isolation in Top Speed mode using CID activation and ion trap detection in each full MS scan with dynamic exclusion enabled. TMT reporter ion quantification was conducted using the Multi-Notch MS3 method[38]; the top5 most abundant MS2 fragment ions with an MS isolation window (m/z) of 0.7 were isolated, following HCD activation and detection using the Orbitrap at a resolution of 60,000, generating an MS3 spectrum.

LC-MS/MS Acquisition Strategy for Crosslinked Peptides. Crosslinked peptides samples resuspended in 5% DMSO / 10% FA / 85% H_2O were separated by a nanoflow LC system (Dionex UltiMate 3000 RSLC nano), utilising the same gradient as above, or an extended 4 h gradient as follow: 0–3 min, 4% B; 3–10 min, 4-8% B; 10-200 min, 8-30% B; 200-210 min, 30-40% B, 210-215 min 40-99%B; 215-219.8 min, 99% B; 219.8-220 min, 99-4% B; 220-250 min, 4% B where A=0.1% FA in H_2O, B=80% ACN, 0.1%FA in H_2O. The nano column (EASY-Spray PepMap® RSLC C18, 2µm 100 Å, 75 *µ*m x 50 cm), set at 40°C was connected to an EASY-Spray ion source (Thermo Scientific). Spectra were collected from an Orbitrap mass analyser (Orbitrap Fusion™ Lumos Tribrid, Thermo Scientific) using full MS mode (resolution of 60,000 at 400 m/z) over the mass-to-charge (m/z) range 375–1600, utilising an XLMS cleavable MS2-MS3 method. Peptides of charge 3-8 were chosen and sorted, favouring highest charged state. Data-dependent MS2 scan was performed using Quadrupole isolation with a cycle time of 5 sec with CID activation and Orbitrap detection at 30,000. A targeted mass difference of Delta M1: 31.9721 was defined to detect the presence of DSSO crosslinked peptides for their analysis in an MS3 scan. Data-dependent MS3 scans were performed using Quadrupole isolation with CID activation and ion trap detection.

MS Database Search and Analysis. Thermo Scientific Proteome Discoverer software (version 2.2.0.388) was used to search non-crosslinked raw data files against the human database (UniProtKB/Swiss-Prot version 2018_02, 20,400 protein entries) using Mascot (version 2.6.0, Matrix Science). The mass tolerance was set at 10 ppm for precursor ions and 0.8Da for fragment ions. Trypsin was used as the digestion enzyme with up to two missed cleavages being allowed. Carbamidomethylation of

cysteines and oxidation of methionine residues were chosen as fixed and variable modifications, respectively. Label-free quantification was conducted through the in-built Minora feature detection node, alongside retention time alignment using the feature mapper node, with a maximum retention time shift of 10 minutes being allowed. Unique and Razor peptide precursor areas were used for quantification and cross-sample normalisation was achieved using the total peptide amount. The in-built TMT 11-plex quantification method was assigned for detection of TMT labels. MS/MS-based peptide and protein identifications were validated with the following filters, a peptide probability of greater than 95.0% (as specified by the peptide prophet algorithm), a protein probability of greater than 99.0%, and at least two unique peptides per protein. TMT-acquired data was again normalised using the total peptide amount. Data was then further scaled using the control pooled sample abundance, correcting for technical variation between injections and TMT groups[39]. XLMS acquired data was searched in Thermo Scientific Proteome Discoverer software (version 2.5) using the third party XlinkX node developed by the Heck lab[6]. The inbuilt MS-cleavable MS2/MS3 DSSO search strategy was used. XLMS raw files for plasma analyses were searched against the human database (UniProtKB/Swiss-Prot version 2020, 20,325 protein entries), while HDL XLMS raw files were searched against an in-house HDL proteome database containing sequence information for 348 proteins. Sequest HT was used with Trypsin as the digestion enzyme with up to two missed cleavages being allowed. Methionine oxidation, DSSO modifications of lysine residues and N-terminal acetylation were set as dynamic modifications, and carbamidomethylation of cysteines was set as a static. MS2_MS3 strategy was set

and the DSSO / +158.004 Da (K) crosslink modification was used while keeping all other settings default. XLMS data visualisation was conducted using xiview.org.

ApoA1 Immunoprecipitation. Goat anti-human apolipoprotein AI polyclonal antibody (AcademyBiomed, TX) was used to isolate HDL from human plasma. The anti-ApoA1 antibody was first validated. Plasma (0.5 μL) was precleared with 50 μL Dynabead™ Protein-G (Thermo Fisher) for 1 hr at room temperature with constant agitation. Precleared plasma was incubated with either anti-ApoA1 or goat IgG (R&D Systems) antibody at stated concentrations in a total volume of 100 μL overnight at 4°C. Antibody complexes were then captured using 50 μL Dynabead™ Protein-G for 1 hr at room temperature with constant agitation. Dynabead™ antibody complexes were washed 3 times with 100 μL PBST (0.02% Tween 20) prior to elution in 100 μL 100 mM glycine, pH 2.0. For large-scale HDL isolation, 1 mg of Goat anti-human apolipoprotein AI polyclonal antibody (AcademyBiomed, TX) was conjugated to cyanogen bromide (CNBr)-activated sepharose following a published protocol[340]. 1 mg of goat IgG (R&D Systems) was conjugated to CNBr-activated sepharose as a negative control. 10 μL of plasma diluted in PBS was incubated with antibody-sepharose resin overnight at 4°C with end-over-end mixing. ApoA1 depleted and bound fractions were isolated as described above.

High-performance Liquid Chromatography Fractionation of Plasma and Lipoproteins. High-performance size-exclusion chromatography was conducted upon healthy pooled volunteer plasma and PCSK9 pre-incubated with lipoproteins. Plasma was separated using a Superose™ 6 Increase 10/300 GL column (Cytiva), 8.6 μm particle size, agarose matrix, 10mm internal diameter. 20 μL of plasma was fractionated with PBS as the mobile phase at a flow rate of 0.6 mL/min collecting a

total of 16 fractions. PCSK9 (20 μg/mL) was pre-incubated with HDL (5 mg/mL), LDL (5 mg/mL) or VLDL (1 mg/mL) overnight at 4°C prior to fractionation. PCSK9-lipoprotein mixes were separated with TSKgel G5000/G4000PWXL- columns (hydroxylated methacrylate, 10 μm particle size, 100 nm mean pore size; Tosoh Bioscience) equipped with a TSKgel. PWXL guard column (hydroxylated methacrylate, 12 μm particle size, mixed pore size; Tosoh Bioscience, 0008033), collecting 16 fractions.

Lipoprotein-associated PCSK9 ELISA. Lipoprotein-associated PCSK9 was measured using an in-house sandwich ELISA. Briefly, microtiter 96-well plates were coated overnight at 4°C with alirocumab (5 μg/mL at 40 μL/well). Excess material was washed off and the plates blocked with 1% Tris-buffered saline/bovine serum albumin for 45 minutes. EDTA plasma was added at 1:50 dilution (40 μL/well) for 75 minutes to allow alirocumab to bind PCSK9. This dilution of plasma provided conditions, whereby a saturating and equal amount of PCSK9 was captured in each well. To detect ApoB-100, Lp(a), or ApoA1 bound to PCSK9 (PCSK9-ApoB, PCSK9-Lp(a), and PCSK9-ApoA1, respectively, biotinylated goat anti-human ApoB-100 antibody (Academy Biomedical Co, Houston, TX) at 1 mg/mL, biotinylated murine monoclonal antibody LPA4 at 1 μg/mL or biotinylated goat anti-human ApoA1 at 0.8 ng/mL, respectively, were added. Alkaline phosphatase-conjugated to NeutrAvidin (Thermo Scientific, Waltham, MA) was added for 60 minutes. Lumi-Phos 530 (Lumigen, Inc, Southfield, Michigan) (25 μL/well) was added for 75 minutes and luminescence read on a Dynex luminometer (Chantilly Technologies, Chantilly, VA). The results are reported as relative light units (RLU) in 100 ms after subtraction of background RLU (Tris-buffered saline/bovine serum albumin blank).

Patient Cohort. From 2006 to 2007, blood samples were prospectively taken from consecutive fasted patients ≥18 years of age presenting either with acute myocardial infarction (MI, ST-segment elevation myocardial infarction or non-ST-segment elevation), stable CAD or microvascular angina at the Clinic of Cardiology, West German Heart Center, University Hospital Essen and the Alfried Krupp Hospital Essen. Microvascular angina was defined as symptomatic patients with coronary stenosis of less than 50%, but with reduced coronary flow reserve as measured by intracoronary Doppler. CAD patients who underwent PCI, were symptomatic of the disease, typically manifesting as angina pectoris, but were ruled out of having MI. Angiographic assessment identified a stenosis of at least 50% in at least one coronary artery in these patients. The study was approved by the Local Ethics Committee (University Hospital Essen, 02-2965). In all groups, 30 mL of blood was drawn into vacuum tubes containing 1.6 mg EDTA/mL (4.298 mM EDTA/L). Immediately after blood drawing, the vacuum tubes were placed on ice and stored at 4°C until further processing. Plasma was generated by centrifugation (1000 x g, 30 minutes, 4°C), immediately recovered and frozen in aliquots at -80°C.

Ultracentrifugation-based Isolation of High-Density Lipoprotein. All experimental procedures were performed by an investigator blinded to patients' data. High-density lipoproteins were isolated by sequential density gradient ultracentrifugation according to their density (1.063–1.21 g/mL), following an established protocol[7]. Protein concentration was determined in each sample by Bradford assay (Bio-Rad, USA). Isolated HDL was then frozen in aliquots at -80°C. The analyses conducted within our study used aliquots that had only undergone one prior freeze/thaw cycle on ice.

Targeted Lipidomics. Lipidomics analysis was conducted using Biocrates AbsoluteIDQ p400 (Biocrates Life Sciences AG) kits according to manufacturer's instructions using both plasma (10 μL) and HDL (10 μg). Plasma and HDL lipid extracts were run by flow injection analysis (FIA), utilising the high resolution, accurate mass of a Q Exactive-Orbitrap MS coupled to a Vanquish Flex UHPLC system (Thermo Fisher), according to the manufacturer's specifications. Raw data was processed using the supplied MetIDQ software. Only Lipids that had a concentration greater than that of the limit of quantification were taken forward for analysis.

HepG2 Cell Culture. The human liver hepatocellular carcinoma cell line, HepG2 (ECACC 85011430), was used as *in vitro* model of cellular cholesterol metabolism. Cells were cultured in Dulbecco's Modified Eagle's Medium (DMEM, Thermo Fisher Scientific) supplemented with 10% heat-inactivated foetal bovine serum (FBS), 2 mM L-glutamine and 1% penicillin/streptomycin (100 U/mL penicillin and 100 μg/mL streptomycin), at 37 °C in a humidified atmosphere of 95% air/ 5% CO_2.

HepG2 Cell Experiments. For PCSK9 studies cells were seeded in 6-well plates at a density of $3x10^5$ per well, and the next day media was changed to DMEM containing 10% lipoprotein deficient serum (LPDS, Merck). 24 h later media was changed and supplemented with stated concentrations of PCSK9 (ACRO Biosystems, PC9-H5223), reconstituted HDL (rHDL, Genway), ultracentrifuge-isolated HDL (ucHDL, Merck), after a prior pre-incubation at 37°C for 1 h to promote PCSK9-HDL interaction, and Actinomycin D (Sigma, A9415) for 6 h. Cellular proteins were isolated by the following; cells were washed twice in ice cold PBS to eliminate secreted protein contamination before the addition of cell lysis buffer (25 mM Tris-HCL, 110 mM NaCl,

2 mM EGTA, 5 mM EDTA, 1% Triton and 0.5% SDS) supplemented with protease inhibitor cocktail (Roche), at pH 7.4. Cells were detached through scraping in cell lysis buffer and full lysis achieved by sonication and lysates were incubated on ice for 30 minutes. Cellular debris was then pelleted by centrifugation, 10,000 x g, for 10 minutes at 4°C. Protein concentration was measured using the BCA protein assay kit (Thermo Fisher).

Cell Surface Protein Isolation. Cell surface proteins were isolated using the Pierce membrane protein isolation kit (Thermo Fisher) according to the manufacturer's instructions. Cells were washed twice with ice-cold PBS before incubation with Sulfo-NHS-SS-Biotin dissolved in PBS (0.25 mg/mL) on an orbital shaker for 30 minutes at 4°C. Membrane protein labelling was stopped using provided quenching solution and cells were scraped and centrifuged at 500 x g for 1 minute and resulting pellets were washed twice with ice-cold PBS. Cells were lysed in lysis buffer supplemented with protease inhibitor (Complete mini-EDTA free Protease inhibitor cocktail, Roche) and proteins were solubilised through sonication; clarified lysates were then incubated with NeutrAvidin agarose for 60 minutes with end-over-end rocking. Membrane proteins were eluted from the NeutrAvidin beads through the incubation with cell lysis buffer containing 50mM DTT.

LDL Uptake Assay. The cellular uptake of LDL was determined using the Image-iT^{TM} Low Density Lipoprotein Uptake Kit, BODIPY-FL, according to the manufacturer's instructions (Thermo Fisher). Cells were seeded in 6-well plates, containing 13mm coverslips (Thermo Fisher), at a density of $3x10^5$ per well, and the next day media was changed to DMEM containing 10% LPDS. After 24 h of cholesterol starvation, cells were treated with either PCSK9 (1 μg/mL), HDL (50 μg/mL) or PCSK9

and HDL for 5 h, in the presence of Actinomycin D (5 μg/mL). Cells were then washed in blank DMEM prior to the addition of 12 μg/mL BODIPY-LDL for 3 h. Cells were then washed three times with PBS containing 0.3% BSA and nuclei were stained using Nuclei, Live Ready Probes reagent (Thermo Fisher), at a working concentration of two drops per 1 mL PBS containing 0.3% BSA.

Immuno-stained coverslips were visualised on a Nikon Spinning Disk confocal microscope, images were acquired using NIS-elements 4.0 software. LDL uptake fluorescent (excitation emission 520 nm) intensity was determined using ImageJ particle analysis. Images were converted to binary and a threshold was manually set, enabling a clear distinction between background and signal of interest, this threshold was then kept constant throughout the analysis. The in-built measure particle function was then used, with a minimum particle size of 5^2 pixels and largest as default infinity. The summed particle intensities for each image were used, and normalised for cell number. The number of nuclei were again calculated using ImageJ, image thresholding and smoothing was done, before using the watershed function to be able to distinguish individual nuclei is tight clusters, lastly particle analysis, with a minimum particle size of 150^2 pixels and largest as default infinity was used.

Statistical Power Calculations. CAD Cohort: Male (n=98) vs Female (n=66) proteomic comparison. Alpha error probability of 0.05 and at a defined power of 0.8, a cohort of this size can confidently detect an effect size d = 0.407. Means: Wilcoxon-Mann-Whitney test. Bruneck Cohort: n=656. Alpha error probability of 0.05 at a defined power of 0.8, a cohort of this size can confidently detect a correlation effect size ρ = 0.109. Correlation: Point biserial model. SAPHIR: n=270. Alpha error probability of 0.05 at a defined power of 0.8, a cohort of this size can confidently detect an effect

size ρ = 0.169. Correlation: Point biserial model. Postprandial Cohorts: n=20. Alpha error probability of 0.05 at a defined power of 0.8. Across all time points: sample size 20, 1 group, 8 measurements can confidently detect an effect size f of 0.217. ANOVA: Repeated measures, within factors. Postprandial Proteomics: Alpha error probability of 0.05 at a defined power of 0.8. Across 3 time points: sample size 8, 1 group, 3 measurements can confidently detect an effect size f of 0.501. ANOVA: Repeated measures, within factors. Power calculations were conducted in G*Power.

Normality Tests. The Shapiro-Wilk test was used to determine whether data conformed to a normal distribution and respective p-values are reported to justify the use of parametric tests where appropriate.

Data regarding Figure 4G: S/N IgG p=0.61, S/N ApoA1 p=0.85, IgG Bound p=0.18 and ApoA1 Bound p=0.64.

Data regarding Figure 6C: PCSK9 p=0.39, PCSK9 + ucHDL p=0.23 and PCSK9 + rHDL p=0.64.

Data regarding 7B: Dimeric PCSK9: PCSK9 p=0.35, PCSK9 + ucHDL p=0.11, anti-HIS: PCSK9 p=0.30, PCSK9 + ucHDL p=0.25 and LDLR: PCSK9 p=0.14, PCSK9 + ucHDL p=0.06.

Data regarding 7D: PCSK9 p=0.48 and PCSK9 + ucHDL p=0.70.

Data regarding 7E: 113 proteins tested within this analysis. 11/113 proteins had a Shapiro-Wilk p-value <0.05. However, due to a low sample size an independent t-test was used.

Statistical Analysis. Proteomic and lipidomic data were filtered to keep only molecules with less than 70% missing values. The remaining missing values were imputed using k-nearest neighbour-imputation method with k equal to 10. The relative

quantities of the molecules were scaled using log_2 transformation. All statistical comparisons have been conducted using non-parametric tests, unless otherwise stated, since normality was not proven for all molecules using Shapiro-Wilk test with p-value threshold of 0.05. The Mann–Whitney U test was used for comparisons between two phenotypes and Kruskal Wallis test for comparisons between more than two phenotypes. The Wilcoxon signed-rank test was used for the non-parametric comparison of two paired groups. The Friedman test with Dunns correction was used upon raw postprandial time course data, comparing the 0 h time point with each other time point. For data visualisation purposes, each individual's concentrations for the measured molecule were made relative to their own 0 h time point. P-values were adjusted using Benjamini Hochberg adjustment for multiple testing when appropriate, keeping proteins with false discovery rate threshold of 5%. Experiment-wide correction for multiple testing was not conducted. Spearman and Pearson correlation, hierarchical cluster analysis and visualisation was conducted in the open-source software Perseus[41]. Hierarchical clustering distance was set to Euclidean and the linkage method used was the average. All other data visualisations were created in Graphpad Prism (Version, 8.2.0, GraphPad Software). Representative images were chosen to best represent the respective mean or median of the grouped data. Schematic diagrams were created using BioRender (Biorender.com).

Online Table III. HDL proteome alterations over the postprandial time course.

Protein ID	0 h mean ± 95% CI	4 h mean ± 95% CI	8 h mean ± 95% CI	p-value	effect size f	effect size d
A1BG	27.68±0.63	28.93±0.65	27.80±0.59	0.0361	0.527	1.117
AFM	26.62±0.23	27.23±0.22	26.80±0.18	0.0083	0.678	1.438
AHSG	29.48±0.28	29.96±0.25	29.58±0.27	0.0454	0.559	1.186
AMBP	28.66±0.13	28.66±0.18	28.21±0.20	0.0100	0.592	1.257
APOC3	31.44±0.22	30.98±0.25	31.00±0.27	0.0302	0.578	1.226
APOC4	25.32±0.42	24.63±0.52	25.24±0.20	0.0377	0.515	1.093
B2M	23.26±0.65	24.44±0.71	22.99±0.79	0.0236	0.596	1.264
C1QA	22.47±0.16	22.83±0.18	22.53±0.22	0.0039	0.638	1.353
C1QB	24.34±0.20	24.17±0.21	23.68±0.23	0.0024	0.727	1.542
CFB	25.53±0.82	27.93±1.18	25.08±0.55	0.0044	0.640	1.358
CFHR1	21.62±0.49	23.65±0.77	21.87±0.56	0.0004	0.730	1.549
F13A1	24.44±0.44	24.63±0.46	23.53±0.27	0.0061	0.698	1.481
FBLN5	19.87±0.19	18.88±0.24	18.70±0.54	0.0005	0.737	1.563
FCGBP	21.07±0.48	21.71±0.35	19.36±0.92	0.0006	0.760	1.613
FCN2	24.21±0.20	23.56±0.29	24.03±0.32	0.0135	0.659	1.398
HPX	26.13±0.19	26.72±0.33	26.10±0.24	0.0127	0.615	1.304
HRG	29.74±0.14	29.26±0.20	29.60±0.22	0.0123	0.628	1.331
ITIH1	30.19±0.12	30.16±0.13	29.77±0.19	0.0090	0.621	1.317
KLKB1	24.59±0.38	25.59±0.44	24.72±0.38	0.0260	0.571	1.210
KNG1	28.40±0.39	29.31±0.50	28.28±0.42	0.0327	0.564	1.197
LGALS3BP	25.96±0.27	25.36±0.31	25.43±0.39	0.0417	0.570	1.210
MASP2	24.59±0.31	24.01±0.17	23.87±0.24	0.0147	0.566	1.201
OAF	23.38±0.47	22.71±0.30	24.09±0.47	0.0047	0.713	1.513
PCSK9	21.89±0.29	21.21±0.43	21.31±0.40	0.0431	0.561	1.190
PROC	25.94±0.24	25.49±0.09	25.42±0.17	0.0153	0.570	1.210
SELENOP	23.43±0.19	23.52±0.19	23.76±0.24	0.0298	0.550	1.166
SERPINA3	25.29±0.43	25.83±0.39	24.97±0.27	0.0090	0.677	1.437
SERPINA6	20.79±0.56	23.29±0.79	21.69±0.51	0.0003	0.828	1.756
SERPINF1	23.02±0.41	23.97±0.61	22.32±0.55	0.0026	0.725	1.537
SERPING1	26.35±0.34	27.41±0.41	26.03±0.60	0.0040	0.695	1.475

*Log2 abundances are represented. The median ± the 95% confidence interval (CI) are shown for each time point (n=8). Significance was determined using the non-parametric Friedman test.

Online Table IV. Sex-driven HDL proteome alterations: TMT quantification.

Protein ID	Male mean ± 95% CI	Female mean ± 95% CI	p-value	q-value	Fold change	Effect Size
ANTXR1	6.32±0.16	6.84±0.17	4.98E-06	1.72E-04	0.59	0.73
APOA1	6.65±0.04	6.67±0.05	2.54E-01	4.37E-01	0.03	0.18
APOA2	6.71±0.06	6.59±0.06	1.66E-03	1.43E-02	-0.11	-0.40
APOA5	5.99±0.19	6.42±0.24	2.15E-03	1.62E-02	0.44	0.45
APOC1	6.65±0.09	6.45±0.13	1.14E-02	4.92E-02	-0.17	-0.34
APOE	6.40±0.14	6.75±0.18	2.35E-03	1.62E-02	0.34	0.47
APOH	6.53±0.16	6.87±0.20	2.92E-03	1.83E-02	0.38	0.46
C4A	6.72±0.16	6.97±0.18	1.88E-01	3.61E-01	0.13	0.17
C4B	6.38±0.12	6.81±0.13	1.42E-05	3.27E-04	0.42	0.69
C5	6.60±0.17	6.33±0.27	1.02E-01	2.52E-01	-0.20	-0.21
C9	6.20±0.14	6.67±0.17	9.80E-04	1.13E-02	0.33	0.47
CLU	6.08±0.17	7.01±0.21	5.55E-07	3.83E-05	0.73	0.79
LBP	6.63±0.13	6.31±0.24	1.60E-03	1.43E-02	-0.52	-0.61
PCSK9	6.64±0.09	6.81±0.12	3.05E-01	5.01E-01	0.11	0.23
PCYOX1	6.40±0.13	6.71±0.15	9.74E-04	1.13E-02	0.35	0.52
PLTP	6.32±0.15	6.90±0.16	2.65E-05	4.57E-04	0.50	0.67
PON1	6.53±0.13	6.82±0.17	3.57E-03	2.05E-02	0.31	0.45
PON3	6.32±0.16	6.64±0.18	6.84E-03	3.15E-02	0.33	0.41
SFTPB	6.39±0.26	5.88±0.27	4.80E-03	2.37E-02	-0.53	-0.42

*Log2 abundances are represented. The median ± the 95% confidence interval (CI) are shown for each group (Male, n=98. Female, n=66). Significance was determined using the non-parametric Mann-Whitney test with Benjamini-Hochberg correction. Proteins listed are those which were significant in either the label-free or TMT analysis.

Online Table V. Sex-driven HDL proteome alterations: Label-free quantification.

Protein ID	Male mean ± 95% CI	Female mean ± 95% CI	p-value	q-value	Fold Change	Effect Size
ANTXR1	22.39±0.23	23.14±0.24	3.00E-06	9.23E-05	0.79	0.67
APOA1	40.97±0.01	40.99±0.02	8.98E-03	4.46E-02	0.02	0.27
APOA2	38.41±0.05	38.19±0.07	5.76E-05	1.27E-03	-0.17	-0.62
APOA5	22.95±0.33	23.65±0.43	7.94E-04	8.15E-03	0.83	0.49
APOC1	34.11±0.11	33.87±0.16	1.57E-02	7.11E-02	-0.21	-0.31
APOE	32.85±0.16	33.28±0.21	7.42E-03	3.81E-02	0.35	0.42
APOH	23.00±0.19	23.45±0.30	9.97E-02	2.37E-01	0.20	0.17
C4A	27.53±0.15	27.98±0.17	1.50E-04	2.35E-03	0.47	0.63
C4B	23.95±0.18	24.37±0.18	2.30E-04	2.95E-03	0.54	0.60
C5	22.29±0.23	21.74±0.35	3.12E-03	1.94E-02	-0.55	-0.40
C9	33.11±0.27	33.87±0.37	3.15E-03	1.94E-02	0.70	0.46
CLU	27.59±0.19	28.63±0.24	9.23E-07	5.07E-05	0.81	0.77
LBP	23.82±0.26	23.41±0.36	2.00E-01	3.81E-01	-0.41	-0.30
PCSK9	23.95±0.08	24.18±0.10	9.29E-04	8.62E-03	0.22	0.55
PCYOX1	27.17±0.16	27.56±0.18	5.49E-04	6.04E-03	0.41	0.50
PLTP	27.81±0.19	28.58±0.21	1.32E-06	5.07E-05	0.74	0.74
PON1	27.03±0.16	27.41±0.21	1.07E-03	9.17E-03	0.46	0.52
PON3	24.99±0.28	25.29±0.30	3.66E-02	1.17E-01	0.43	0.31
SFTPB	25.38±0.32	24.85±0.39	5.57E-02	1.62E-01	-0.49	-0.31

*Log2 abundances are represented. The median ± the 95% confidence interval (CI) are shown for each group (Male, n=98. Female, n=66). Significance was determined using the non-parametric Mann-Whitney test with Benjamini-Hochberg correction. Proteins listed are those which were significant in either the label-free or TMT analysis.

Online Table VI. HepG2 cell surface protein changes upon PCSK9 and ucHDL treatment.

Gene Symbol	CTRL ± 95% CI	PCSK9 ± 95% CI	ucHDL ± 95% CI	CTRL/PCSK9 p-value	CTRL/ucHDL p-value
SDC2	21.31±0.02	21.12±0.06	21.18±0.02	6.55E-03	1.41E-03
SDC1	21.89±0.02	21.73±0.07	21.65±0.07	1.98E-02	4.40E-03
LDLR	19.44±0.40	18.69±0.13	19.48±0.31	2.68E-02	8.78E-01
APP	18.74±0.12	18.32±0.22	18.63±0.24	3.29E-02	4.55E-01
LAMP1	25.85±0.02	25.80±0.03	25.77±0.01	4.95E-02	3.50E-03
AIMP1	24.48±0.01	24.58±0.06	24.73±0.01	5.49E-02	2.30E-05
APOH	24.05±0.02	23.98±0.08	23.87±0.02	2.02E-01	4.35E-04
DMD	20.10±0.06	19.90±0.18	19.86±0.05	1.11E-01	4.64E-03
TLN1	23.92±0.01	23.79±0.11	23.82±0.02	1.02E-01	5.17E-03
VAMP3	23.97±0.06	23.87±0.06	23.71±0.08	6.97E-02	7.20E-03
TNFRSF10B	21.53±0.01	21.52±0.05	21.66±0.04	5.96E-01	8.86E-03
IDE	22.10±0.04	22.17±0.05	22.21±0.01	1.37E-01	1.05E-02
HMGB1	25.69±0.05	25.63±0.15	25.26±0.17	5.56E-01	1.09E-02
FGB	20.54±0.05	20.30±0.29	20.37±0.05	1.84E-01	1.16E-02
PLXNB2	23.68±0.03	23.61±0.06	23.56±0.05	1.23E-01	1.99E-02
PHB2	28.91±0.08	28.81±0.23	28.69±0.08	4.78E-01	2.17E-02
TFPI	23.63±0.15	23.86±0.05	23.94±0.06	5.09E-02	2.23E-02
ENO1	26.92±0.08	26.85±0.20	26.65±0.13	5.73E-01	2.79E-02
ANO6	23.31±0.07	23.24±0.09	23.15±0.04	3.72E-01	2.97E-02
PSEN1	20.74±0.06	20.72±0.03	20.61±0.05	5.36E-01	3.20E-02
APOA1	26.82±0.10	26.85±0.02	27.03±0.10	6.17E-01	4.52E-02
ADAM17	19.88±0.17	20.07±0.13	20.20±0.14	1.45E-01	4.82E-02

*Log2 abundances are represented. The mean ± the 95% confidence interval (CI) are shown for each group (n=3). Significance was determined using an independent t-test. Protein listed were significant in at least one comparison to CTRL group.

Online Figure I

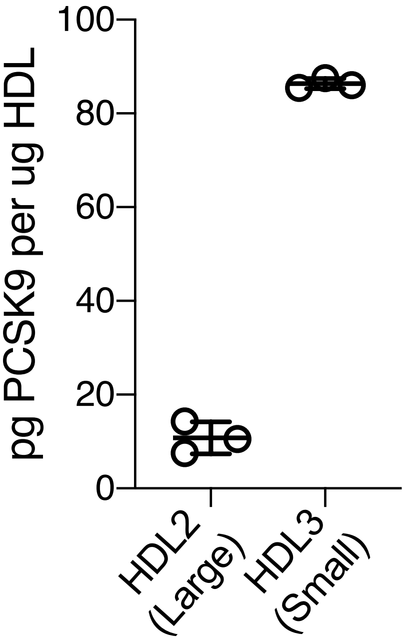

Online Figure I. PCSK9 is enriched in small-HDL. The PCSK9 content of pooled HDL2 (1.063-1.125 g/mL) and HDL3 (1.125-1.210 g/mL) subfractions, obtained from a commercial source, was determined using an anti-PCSK9 ELISA. 10 ug of both HDL2 and HDL3 was used as the input for this assay and each subfraction was run in triplicate.

Online Figure II

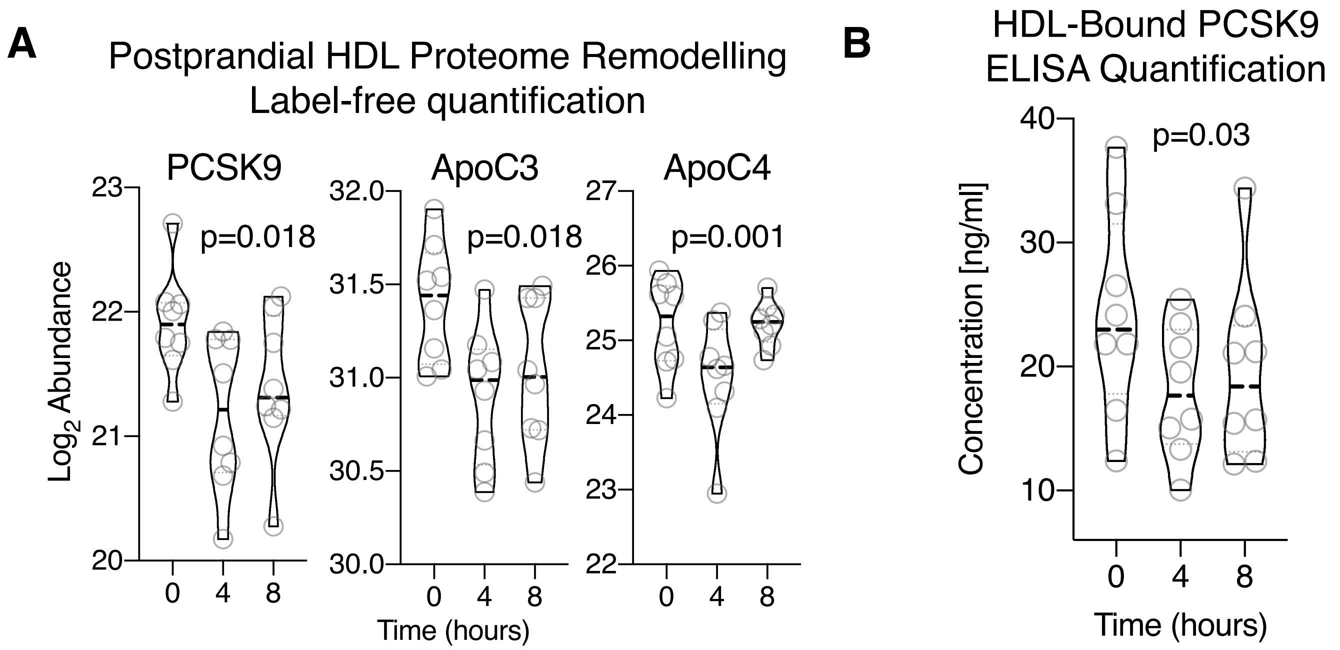

Online Figure II. Postprandial HDL proteome remodelling. Label-free proteomics was conducted upon HDL immuno-isolated from postprandial plasma samples (n=8, 3 times points). **A**, PCSK9, ApoC3 and ApoC4 are separately plotted. **B**, The postprandial change in HDL-bound PCSK9 was validated using an ELISA, significance was determined using the non-parametric Friedman test with Dunn's correction.

Online Figure III

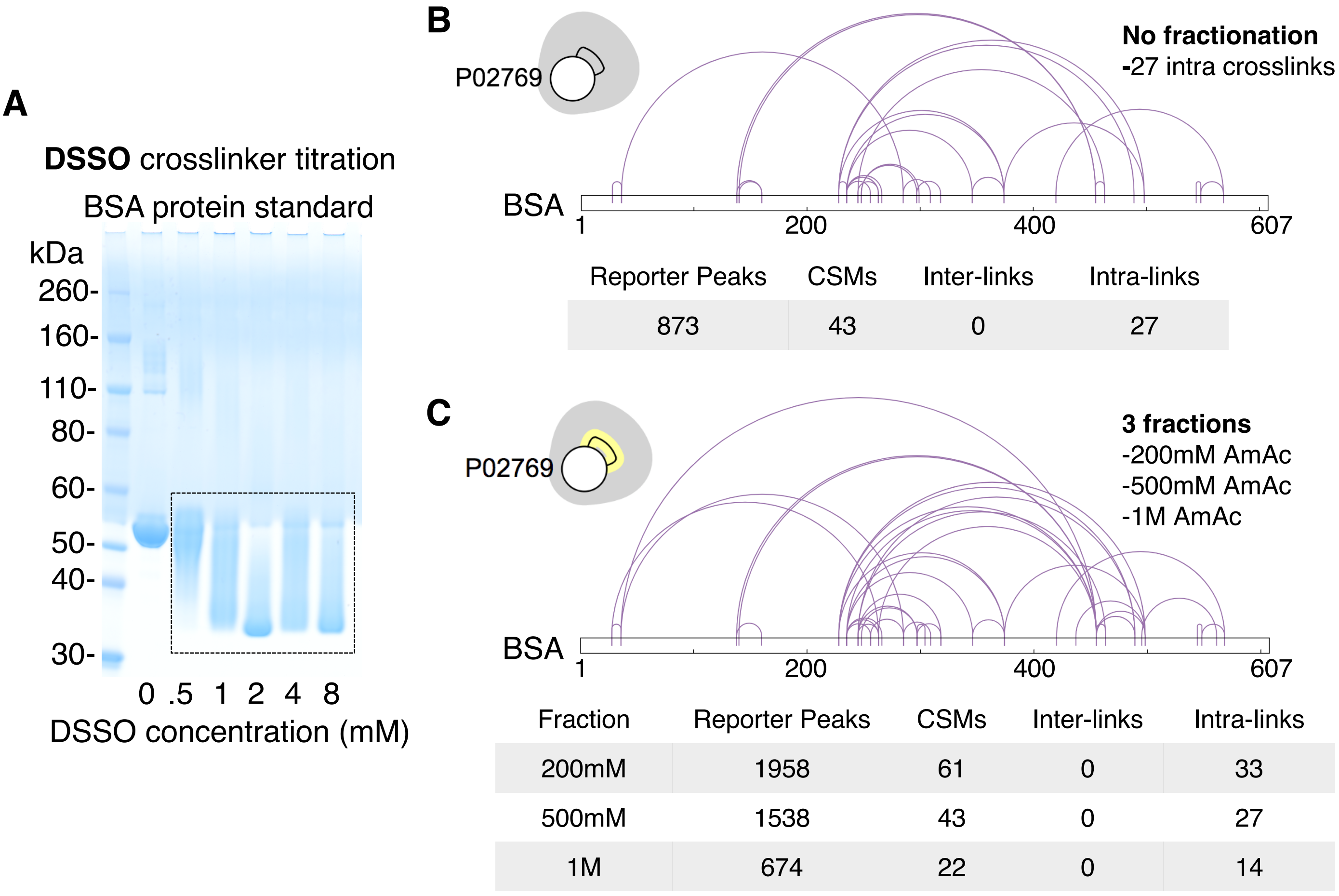

Reporter Peaks	CSMs	Inter-links	Intra-links
873	43	0	27

Fraction	Reporter Peaks	CSMs	Inter-links	Intra-links
200mM	1958	61	0	33
500mM	1538	43	0	27
1M	674	22	0	14

Online Figure III. XLMS methodological development using BSA. The optimal concentration of the DSSO crosslinker was determined through a titration-based experiment and the total protein is visualised on a gel, a downward shift is primarily seen due to intra-molecular crosslink formation (**A**). BSA was crosslinked using a final concentration of 1 mM DSSO and analysed by XLMS without (**B**) and with (**C**) prior strong cation exchange peptide fractionation.

Online Figure IV

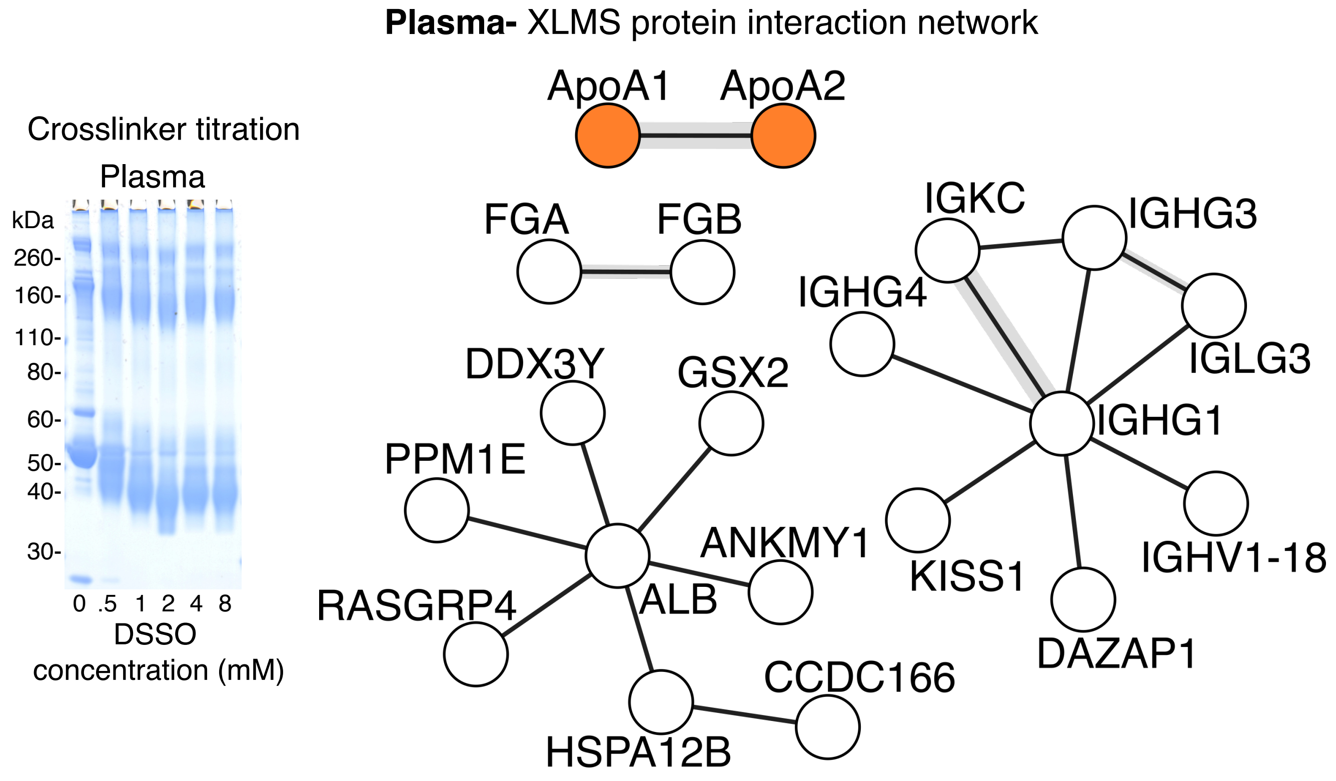

Online Figure IV. XLMS plasma protein interactome. Titration of the MS-cleavable crosslinker DSSO was conducted to determine optimal DSSO concentration, resulting in 1 mM to be used for subsequent analyses. Plasma (n=8 healthy subjects, pooled) was analysed by XLMS and identified inter-protein crosslinks are represented as an interaction network (xiview.org).

Online Figure V

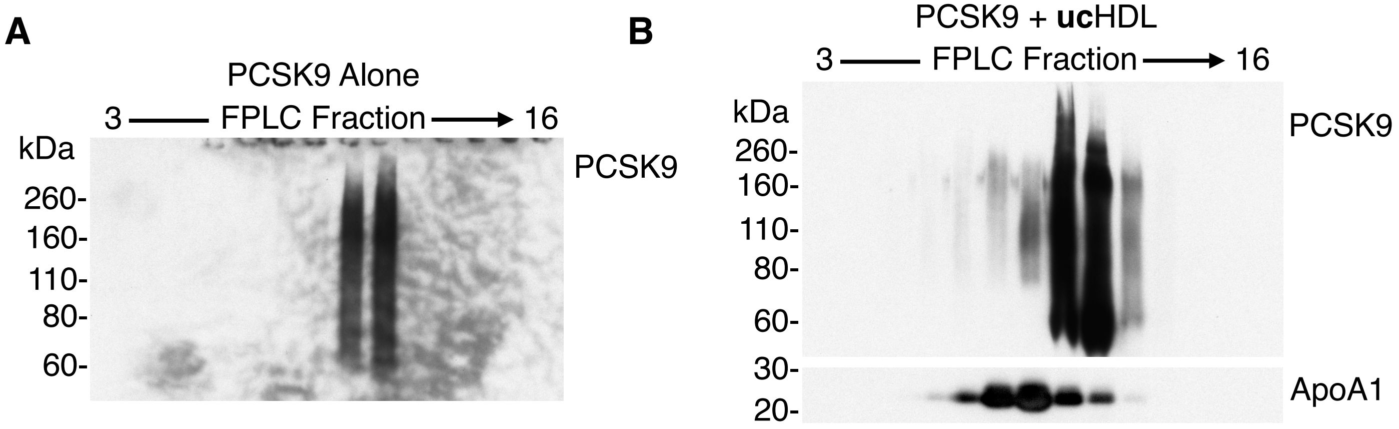

Online Figure V. PCSK9 and HDL interaction. Recombinant HIS-tagged PCSK9 (20 µg/mL) was incubated alone (**A**) or with ultracentrifuge isolated HDL (5 mg/mL) (**B**) overnight at 4°C prior to size exclusion chromatography separation (Superose™ 6 10/300 GL). PCSK9 and ApoA1 abundances were visualised by western blot analysis.

Online Figure VI

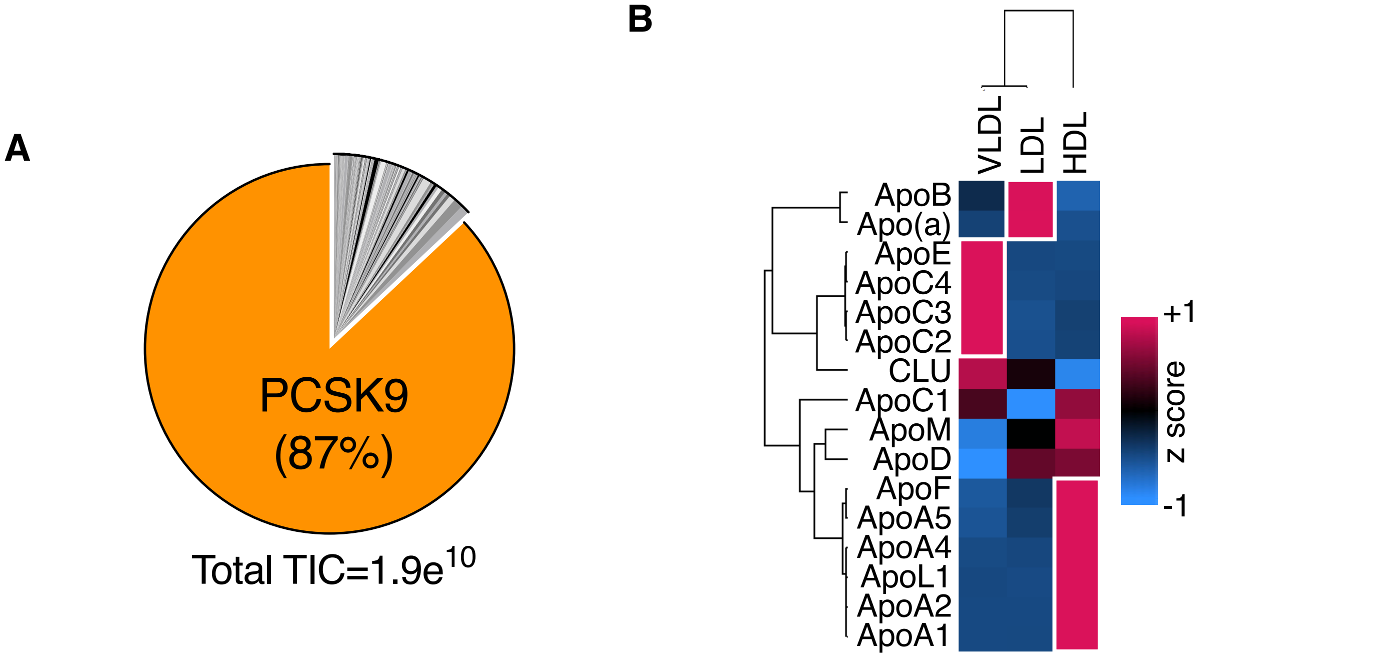

Online Figure VI. MS-based validation of PCSK9 and lipoproteins used for experiments. Commercially-sourced recombinant HIS-tagged PCSK9 and lipoproteins isolated by ultracentrifugation were analysed by MS. **A**, The purity of recombinant PCSK9 was determined through the measure of PCSK9's contribution to total ion current (TIC). **B**, The apolipoprotein profiles of HDL, LDL and VLDL were compared to confirm expected distributions and therefore purity of lipoprotein fractions.

Online Figure VII

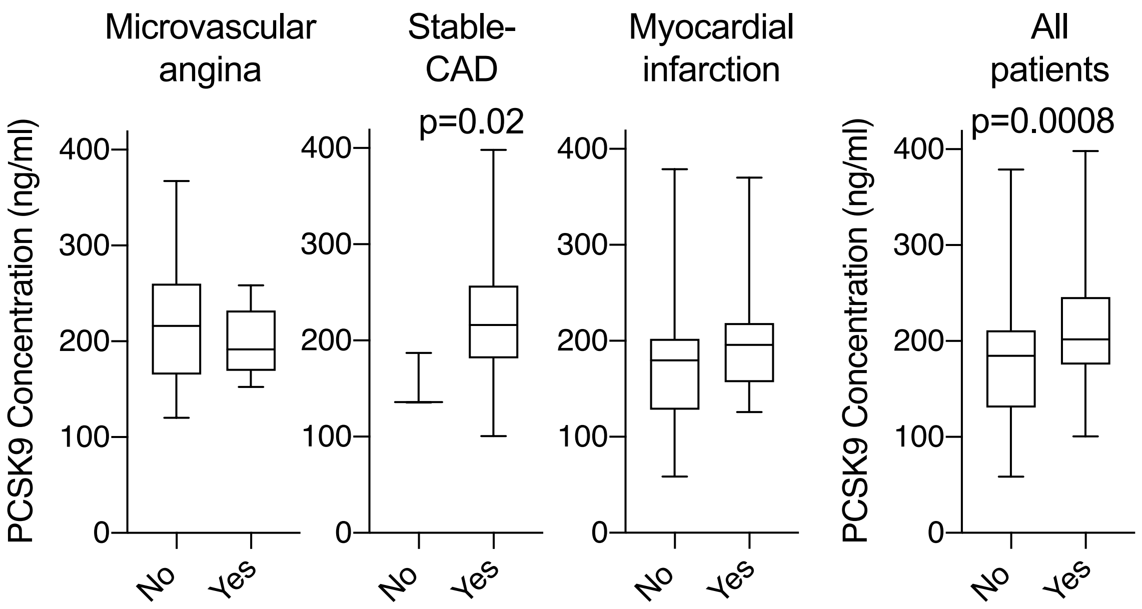

Online Figure VII. PCSK9 plasma levels in response to statin treatment. Statins are known to increase circulating PCSK9 levels. The effect of statins upon plasma PCSK9 concentrations across our CAD cohort is shown above. PCSK9 plasma concentrations were determined by ELISA. Significance was determined by the Mann-Whitney test.

Online Figure VIII

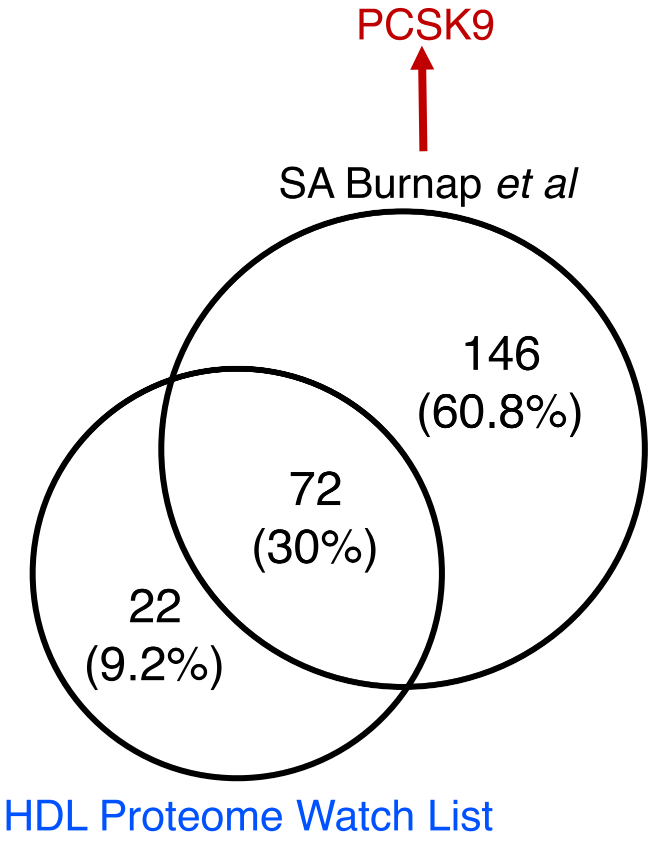

Online Figure VIII. Proteomic analysis of HDL: Comparison against published reports. The HDL proteome watch list is a meta-analysis by the Sean Davidson lab (http://homepages.uc.edu/~davidswm/HDLproteome.html), that compiles identified proteins associated with HDL in studies using high-resolution mass spectrometry based techniques. HDL proteins identified in our study that overlap with proteins identified in at least 3 previous independent studies out of the 17 studies analysed in the meta-analysis conducted by the Davidson lab are represented in a Venn diagram.

Online Figure IX

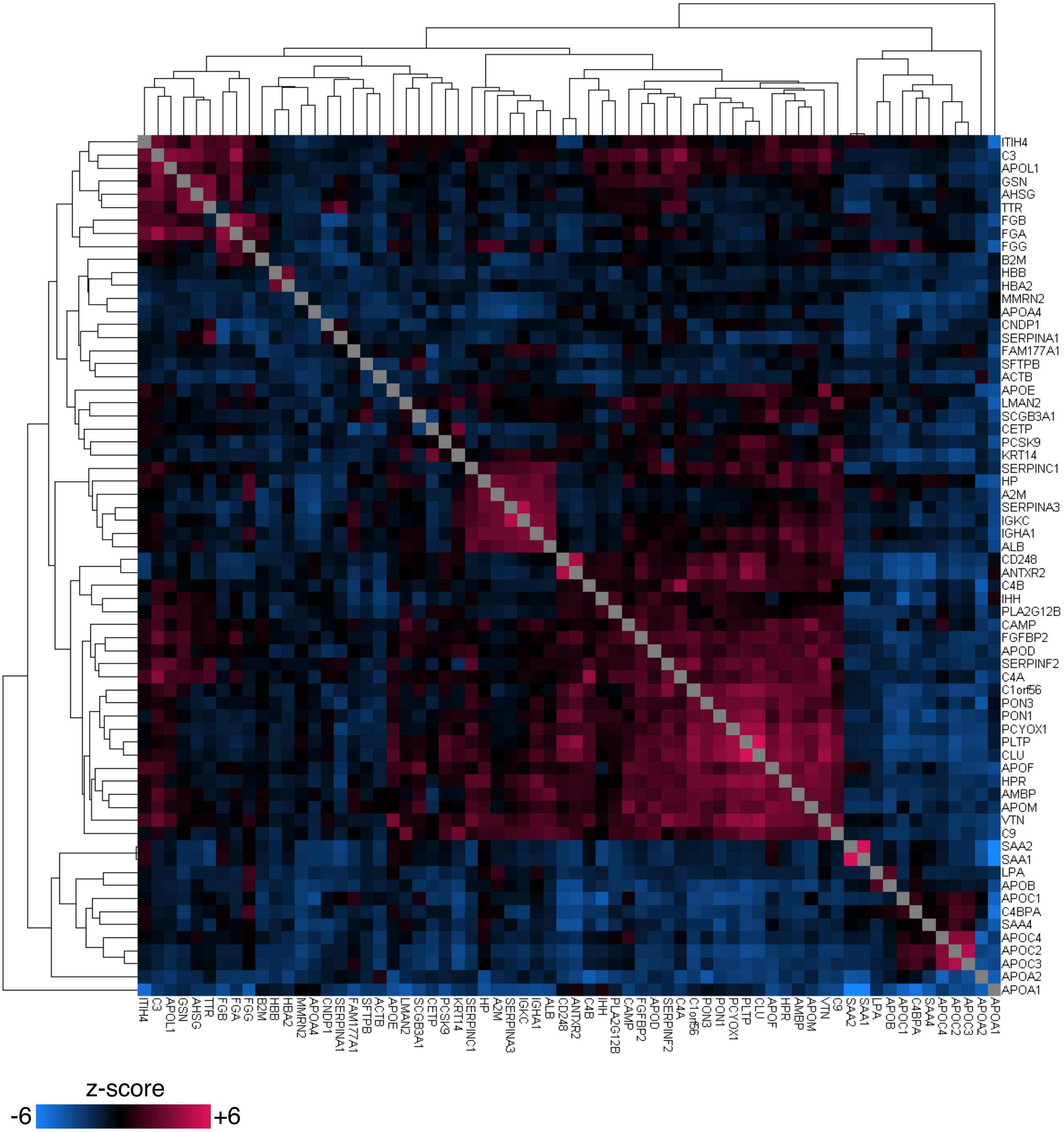

Online Figure IX. HDL proteome correlations. A Pearson correlation matrix was generated for proteins quantifiable in every HDL sample across the cohort (66 proteins including PCSK9) and a hierarchical cluster analysis was conducted upon the resulting matrix, being represented in heat map form.

Online Figure X

**APOA1** 3.67	**APOA2** 3.43	APOA4 2.19	APOB 1.99	APOC1 2.87	CETP 0.36	C3 1.45	C4BPA 1.03	SERPIN A1 2.65	SERPIN A3 0.89	SERPIN C1 0.68
APOC2 2.39	APOC3 3.18	APOC4 1.73	APOD 2.21	APOE 2.55	**PCSK9** 0.78	C4A 1.13	C9 1.61	PCYOX1 1.69	PLA2G12 B 1.20	SERPIN F2 1.34
APOF 1.84	APOL1 1.98	APOM 2.58	**CLU** 1.65	LPA 1.28	**PLTP** 1.65	C4B 1.18	VTN 1.22	PON1 1.54	PON3 1.49	C1orf56 1.07
ANTXR2 1.35	CAMP 1.95	ITIH4 1.12	**SAA1** 2.57	A2M 0.8	AHSG 1.2	ALB 2.2	AMBP 1.1	CD248 0.71	CNDP1 1.05	FAM177A 1 0.68
**SAA2** 2.30	SAA4 3.03	SCGB3A1 1.57	**SFTPB** 1.39	B2M 0.9	HBA2 1.6	HBB 1.6	HP 1.7	FGFBP2 1.27	GSN 0.77	IHH 1.12
ACTB 0.86	FGA 1.66	FGB 1.39	FGG 1.17	HPR 1.8	IGHA1 1.5	IGKC 1.8	TTR 2.1	KRT14 0.04	LMAN2 0.89	MMRN2 0.63

Online Figure X. PCSK9 is a member of the HDL proteome in patients with CAD. HDL was isolated from 172 patients with varying CAD-related phenotypes by density-ultracentrifugation. Proteins quantifiable in all HDL samples by label-free MS were considered as the core HDL proteome (n=191, including patients with 6 months follow-up). Proteins are grouped based on Reactome pathway analysis and functionality. Orange = Apolipoproteins, Pink = Lipid Metabolism, other colours represent related protein clusters. Number represents total Peptide Spectrum Matches / Molecular Weight (PSM/mW). Colours are obtained by conditional formatting.

Online Figure XI

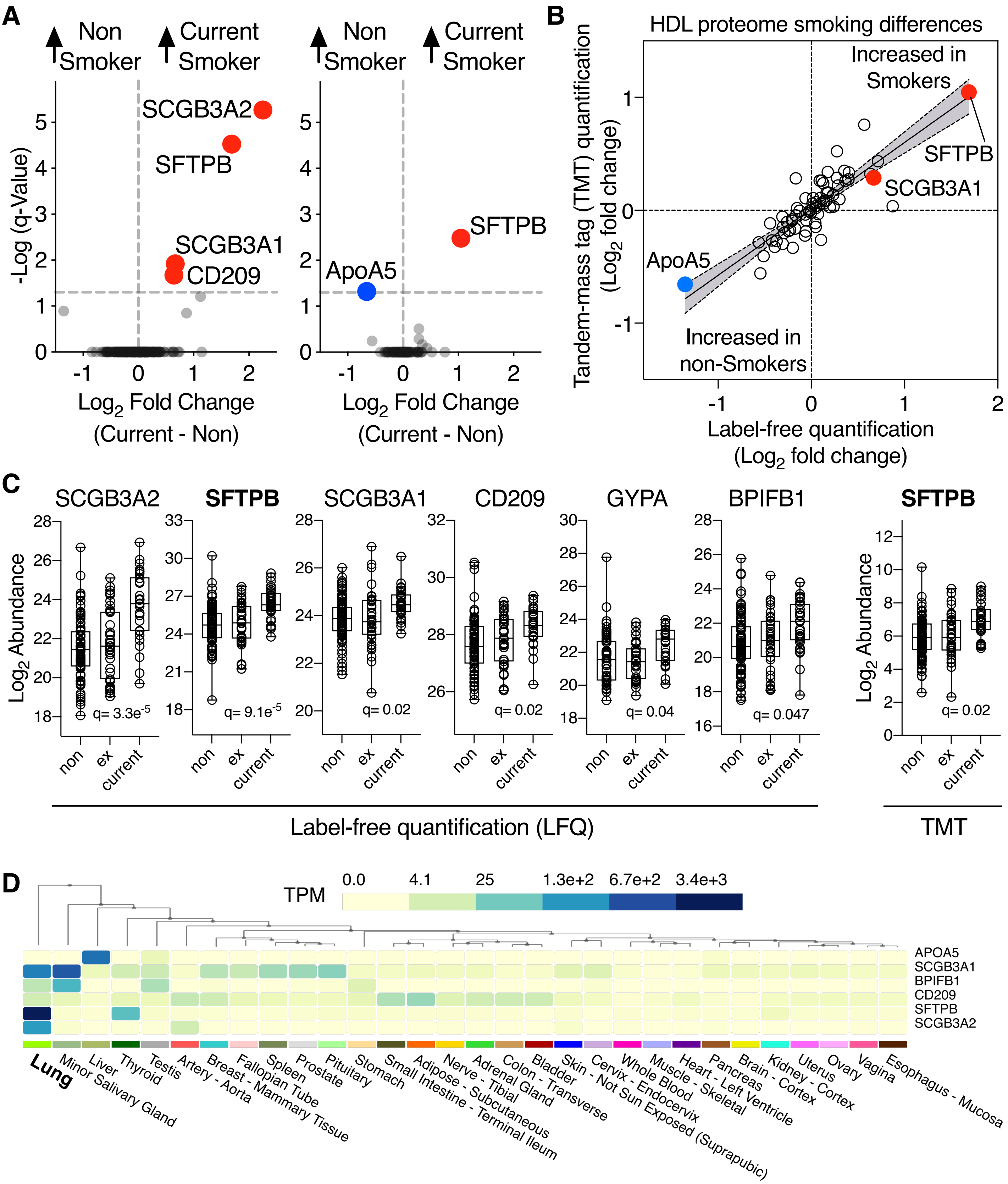

Online Figure XI. Smoking drives HDL proteome alteration. A, B, Label-free and TMT-based proteomics was used to determine HDL proteome alterations in response to smoking within the CAD cohort. Protein changes across current smokers compared to non-smokers are represented. **C,** Protein changes were not evident in ex-smokers. **D,** Proteins enriched in the HDL proteome of smokers were primarily lung-derived (https://gtexportal.org/).

Online Figure XII

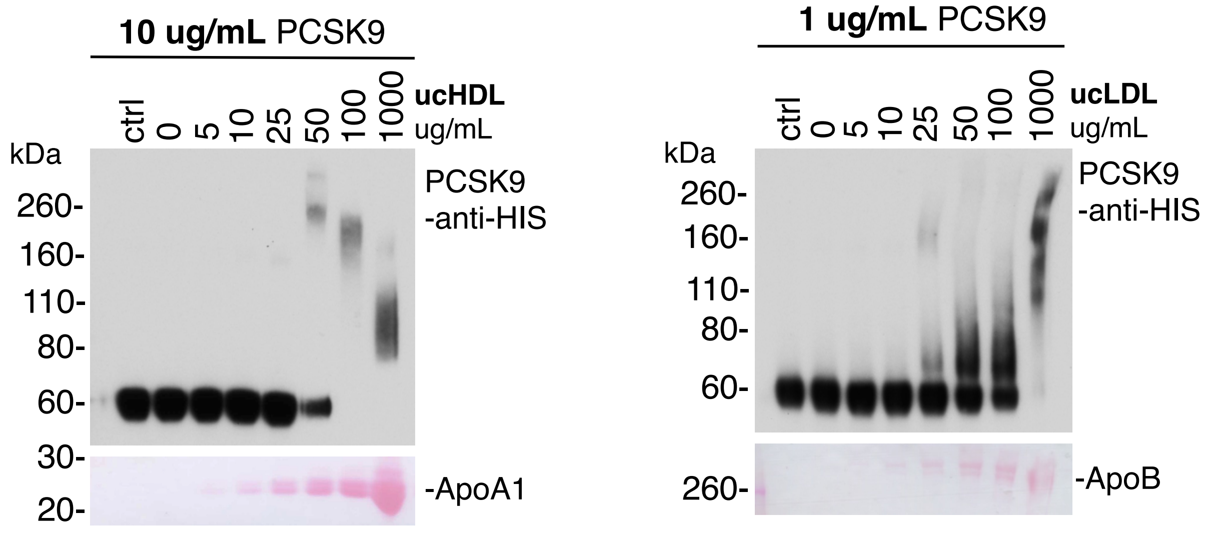

Online Figure XII. HDL and LDL differentially induce PCSK9 multimerisation. Recombinant His-tagged PCSK9 at a concentration of either 10 ug/mL or 1 ug/mL was incubated in the presence of an increasing concentrations of ucHDL (**A**) and ucLDL (**B**) for 24hrs at 37°C. The control lane represents PCSK9 incubated without lipoproteins at 4°C for 24hrs. Immunoblot analysis was then conducted, with equal amounts of PCSK9 loading for each sample. Total protein stain is used to visualise ApoA1 and ApoB.

Online Figure XIII

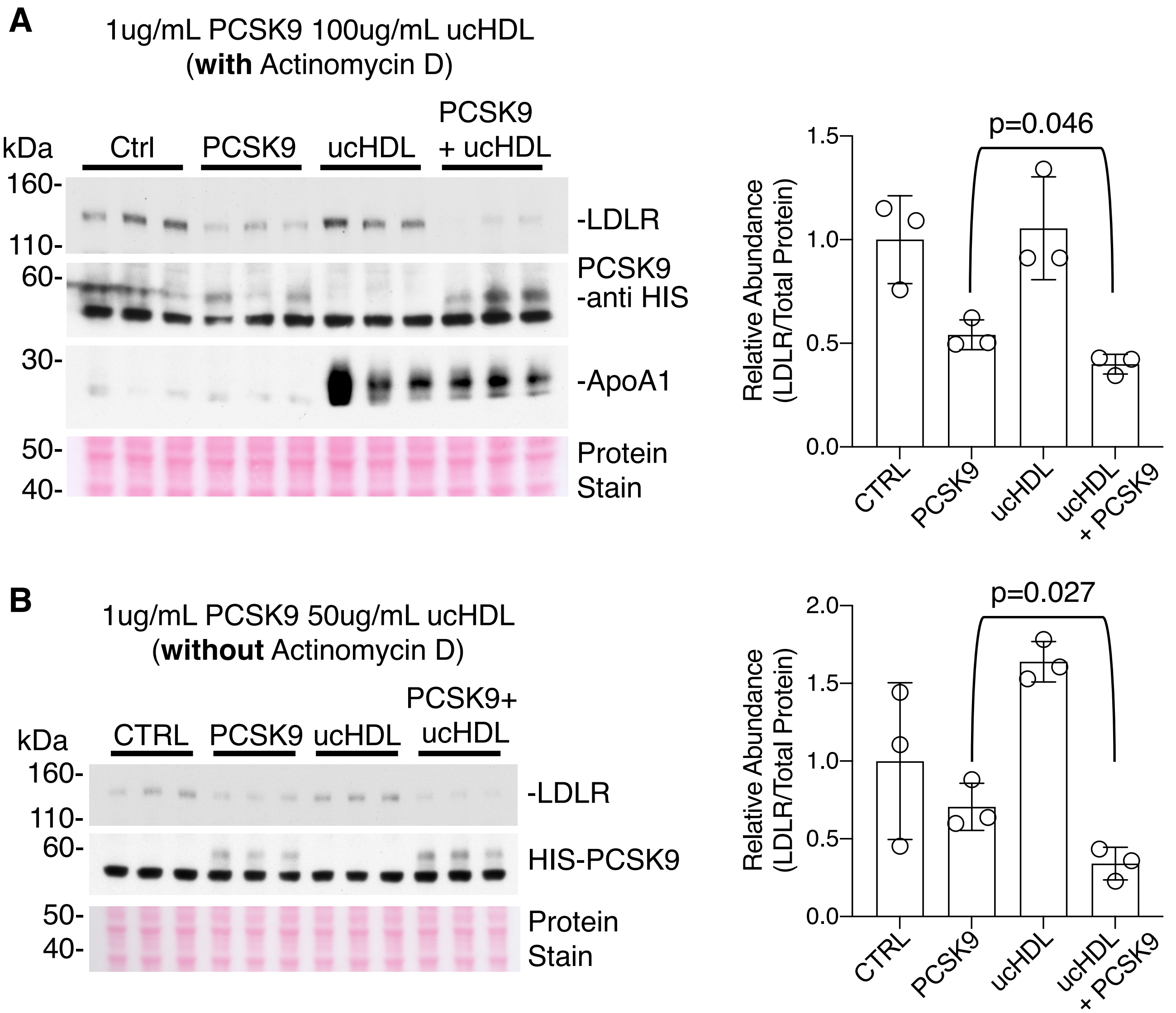

Online Figure XIII. HDL potentiates PCSK9-mediated LDLR degradation. A, HepG2 cells were treated with HIS-tagged PCSK9 (1 μg/mL), ucHDL (100 μg/mL) or a combination of ucHDL and HIS-tagged PCSK9 for 6 h in the presence of actinomycin D (5 μg/mL), prior to immunoblot analysis. **B,** HepG2 cells were treated with HIS-tagged PCSK9 (1 μg/mL), ucHDL (50 μg/mL) or a combination of ucHDL and HIS-tagged PCSK9 for 6 h without actinomycin D, prior to immunoblot analysis. Significance was determined using an independent t-test.

Online Figure XIV

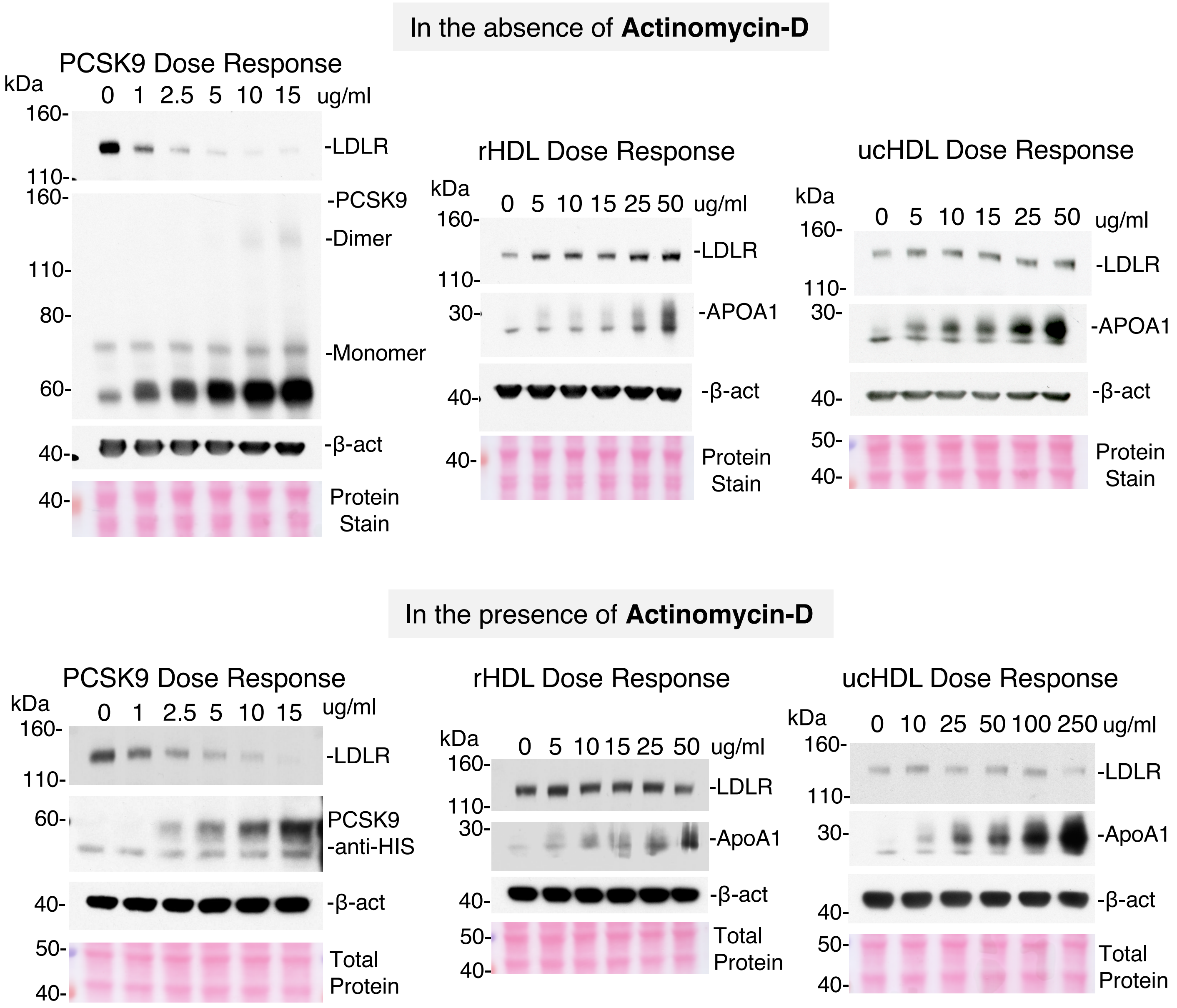

Online Figure XIV. Actinomycin D ablates HDL-mediated LDLR synthesis. HepG2 cells were treated with increasing doses of rHDL, ucHDL and His-tagged PCSK9 for 6hrs, without or in the presence of actinomycin-D (5 ug/ml), followed by immunoblot analysis.
